# *Selenicereus undatus* (Dragon Fruit) Phytochemicals for Managing Three Human Pathogenic Bacteria: An In Vitro and In Silico Approach

**DOI:** 10.3390/metabo14110577

**Published:** 2024-10-25

**Authors:** Zhuan-Ying Yang, Xue-Wen Zheng, Wen-Hao Jiang, Gui-Zhi Chen, Qing-Zhi Liang, Guang-Zhao Xu, Run-Hua Yi

**Affiliations:** College of Coastal Agricultural Sciences, Guangdong Ocean University, Zhanjiang 524088, China; irene0411@gdou.edu.cn (Z.-Y.Y.); 2112104035@stu.gdou.edu.cn (X.-W.Z.); 2112204052@stu.gdou.edu.cn (W.-H.J.); 202111321102@stu.gdou.edu.cn (G.-Z.C.); qingzhi2002@gdou.edu.cn (Q.-Z.L.)

**Keywords:** dragon fruits, antibacterial, metabolite, molecular docking, molecular dynamics simulation

## Abstract

Objectives: Antibiotic-resistant bacterial infections are a growing global concern. A natural remedy for bacterial infections could be available in the *Selenicereus undatus* fruit, but its antibacterial and biochemical properties are not fully known. Methods: In this study, the biochemical composition and antibacterial, antioxidant, and cytotoxic activities of the Jindu No. 1 (JD) and Bird’s Nest (YW) dragon fruit varieties and their potential effects against *E. coli*, *Pseudomonas* sp., and *Staphylococcus* sp. were scrutinized. Results: The JD fruit extract showed higher antibacterial activity than the YW variety against *E. coli*, *Pseudomonas* sp., and *Staphylococcus* sp. in vitro. Additionally, the JD variety demonstrated more significant antioxidant activity than the YW variety and showed less cytotoxic activity. The JD variety had a higher glucose content, while the YW variety had a higher fructose content, and the phytoconstituents analysis confirmed 659 metabolites in total from the two varieties. Through in silico analyses, phytoconstituents were evaluated to identify potential drug molecules against the selected bacterial strain. Moreover, the molecular docking study revealed that riboprobe and Z-Gly-Pro might be effective against *E. coli*, 4-hydroxy retinoic acid, and that succinyl adenosine may target *Pseudomonas* sp., and xanthosine and 2′-deoxyinosine-5′-monophosphate may be effective against *Staphylococcus* sp. These results were further validated by 100 ns Molecular Dynamics (MD) simulation, and all of the selected compounds exhibited acceptable ADMET features. Conclusions: Therefore, phytoconstituents from *S. undatus* fruit varieties could be employed to fight human bacterial diseases, and future studies will support the continuation of other biological activities in medical research.

## 1. Introduction

*Selenicereus undatus* is commonly known as dragon fruit in southeast Asia and extensively grown in southern China [1,2]. It is a prevalent fruit due to its peculiar look, nutritional value, and delicate texture [3,4,5]. It is commercially grown in many countries throughout the globe, including China, Thailand, Colombia, Mexico, Malaysia, Vietnam, Australia, Nicaragua, Indonesia, Ecuador, USA, etc. [6,7]. It is becoming famous in Asian countries because of its soluble proteins, sugars, and minerals [8]. It contains a variety of phytochemicals, including flavonoids, fiber, vitamins B complex (B1, B2, and B3), phosphorus, calcium, and polyphenols, and has antioxidant capacity that is beneficial for human health [9,10]. Flavonoids help to cure cardiovascular disease, fiber assists in digestion, vitamin B1 assists in carbohydrate metabolism and energy production, vitamin B2 helps to improve and recuperate from appetite loss, vitamin B3 helps to decrease harmful cholesterol levels, phosphorus and calcium strengthen bones and teeth, and anticancer properties play an essential function in tissue generation [11,12,13,14,15,16,17]. *S. undatus* is also beneficial in lowering blood sugar levels in those with type 2 diabetes; research shows that the glucose in dragon fruit aids in diabetes patients’ blood sugar control. These compounds function in the human body as antioxidative, anti-inflammatory, radioprotective, antiproliferative, cardioactive, diuretic, hypoglycemic, and hypolipidemic molecules and pain relievers for osteoarthritis. Additionally, it can exhibit decisive antibacterial action against *Bacillus* sp., *Staphylococcus* sp., *Salmonella* sp., *Klebsiella pneumonia*, *Enterococcus faecalis*, *Escherichia coli*, *Yersinia enterocolitica*, and *Pseudomonas* sp. [18,19,20]. Dragon fruit may directly or indirectly produce a range of metabolites with various biological activities to provide humans with sustenance, energy, and drugs [21,22,23].

Microbial species disturb normal body functions and lead to infectious diseases that often possess complex structural or physiological characteristics, contributing to antibiotic and drug resistance [24,25]. *Streptococcus* sp. has pathogenicity attributes that contribute to skin and respiratory infections [26], whereas urological and gastrointestinal diseases are often associated with *E. coli* and *Pseudomonas* sp. [27]. The widespread use of antibiotics is the primary cause of infectious microorganism resistance to treatment [28]. According to the World Health Organization (WHO), approximately 50,000 individuals lose their lives daily as a result of infections resistant to antibiotics [29]. Additionally, traditional medicine is relied upon by 65% to 80% of the global population [30]. Local plants and fruits could help fight infectious diseases, and phytoconstituents are of great interest to researchers seeking new drugs to treat antibiotic-resistant microorganisms [31]. Numerous plants possessing antioxidant properties offer positive health benefits [32]. Oxidative stress caused by free radicals in the body is implicated in the mechanisms of cancer, atherosclerosis, and diabetes, as well as neurodegenerative and inflammatory diseases [33,34]. Antioxidants play a role in mitigating oxidation by scavenging free radicals and reducing oxidative stress [35,36]. Several secondary metabolites in plants exhibit antimicrobial properties such as alkaloids, phenols, flavonoids, and tannins [37].

This investigation aimed to study the antibacterial and biochemical profiles, antioxidant and cytotoxic activity, and sugar content of the *S. undatus* varieties under laboratory conditions. The chief goal of this study was to screen *S. undatus* phytochemicals for their potential efficacy in treating three human pathogenic bacteria. In-depth in silico analysis was employed to identify potential compounds in *S. undatus* that can interact with the virulence proteins of *E. coli*, *Pseudomonas* sp., and *Staphylococcus* sp., thereby inhibiting their activity. This investigation sheds light on the promising medicinal properties of the *S. undatus* fruit, showcasing its potential as a source of antibacterial and antioxidant agents. The use of in silico techniques adds a computational dimension to the study.

## 2. Materials and Methods

### 2.1. Sample Collection and Extract Preparation

**Figure 1:**
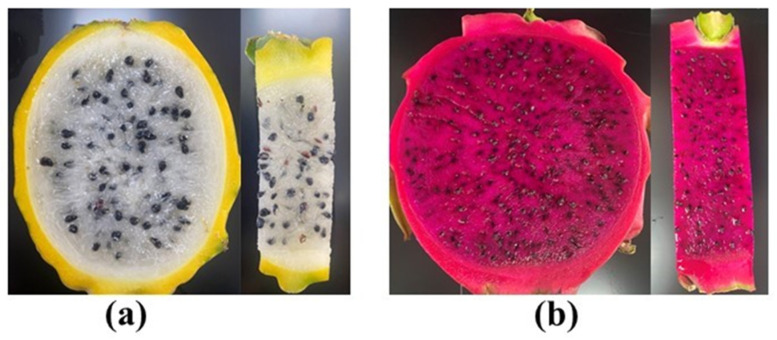
Skin and pulp color differences between two dragon varieties. Here, (**a**) indicates the YW variety and (**b**) indicates the JD variety.

This experiment was conducted on two *S. undatus* varieties during the fruiting season. Jindu No. 1 (characterized by reddish skin and flesh; denoted as JD) and Bird’s Nest (characterized by yellowish skin and white flesh; denoted as YW) fruit samples were collected from Guangdong Ocean University, China (long. 110°30″ E and 21°15″ N) (Figure 1a,b). The fruit samples were aseptically transferred in frozen liquid nitrogen. Strains of the bacteria *E. coli*, *Staphylococcus* sp., and *Pseudomonas* sp. were acquired, and further biochemical analyses were carried out at the Tropical Crop Efficient Production Engineering Technology Research Center, Binhai Agricultural College, Guangdong Ocean University. The disease-free samples were sliced and transferred into an incubator at 65 °C for proper drying and then powdered using an electric grinding machine. The methanolic extracts were prepared according to Mariswamy et al. [38], and 25 g of powdered samples were added to 100 mL solvents and continuously shaken for seven days on an orbital shaker in vials covered with aluminum foil and parafilm. Then, the mixture was filtered using Whatman no. 1 filter paper for proper filtration. Finally, the filtered samples were kept at room temperature for proper evaporation.

### 2.2. Antibacterial Activity Test

Antibacterial activity was evaluated using the methodology described by Nostro et al. [39]. First, 6 mm discs were prepared using the Whatman No. 1 filter paper and sterilized. Then, both extract’s final concentrations were adjusted to 5 μg/mL and the discs soaked with diluted extract at concentrations of 50, 100, and 150 μg/mL. Finally, 150 μL of each overnight bacterial culture was transferred onto each agar plate, and the treated discs were placed on each plate with sterile forceps. Kanamycin was used as a control. Then, the plates were kept overnight at 37 °C for observation, and inhibition zones were measured using a millimeter (mm) scale.

### 2.3. Antioxidant Activity Test

Antioxidant activity was tested by following the method described by Rahman et al. [40] using a DPPH-free radical scavenging assay. For this, each solvent extract concentrations were adjusted to 50, 100, 150, 200, and 250 μg/mL and made up to a volume of 1 mL by adding methanol. The total volume was then made up to 2.5 mL by adding 1.5 mL of DPPH (0.1 mM) solution to each vial under dark conditions for 30 min. Following a half-hour, optical density (OD) was assessed at 517 nm using a spectrophotometer. The BHT standard curve was prepared according to the method described by Jahan et al. [41]. The scavenging percentage was calculated according to the method described by Mahmud et al., where the half maximal inhibitory concentration (IC_50_) value was calculated by plotting the equation in an Excel linear scatter graph [42].

### 2.4. Cytotoxic Activity Test

The cytotoxic activity of these extracts was evaluated through brine shrimp nauplii (*Artemia salina*) according to the method described by Meyer et al. [43]. For this, *Artemia salina* was hatched for 48 h in artificially prepared 1% NaCl seawater. The concentrations of each extract were adjusted to 25, 50, 100, 150, and 200 μg/mL. Then, 15 live brine shrimp were placed in each vial with 5 mL of artificial seawater and kept for 24 h at room temperature. After 24 h, the total deaths and lives were counted. Finally, the 50% lethal concentration (LC_50_) value was calculated by plotting the equation y = mx + c in an Excel linear scatter graph, where y = 50, x = unknown concentration, m = coefficient of x, and c = constant.

### 2.5. Biochemical Characterization

#### 2.5.1. Sugar Content Analysis

The total sugar content (including fructose, glucose, and lactose) was determined according to the methodology outlined by Liu et al. [44]. In this procedure, a 1.0 g pulp sample was subjected to microwave heating for 30 s at 100 °C to inactivate enzymes. The sample was subsequently homogenized in distilled water using mortar and pestle. A 2-milliliter diluted sample was collected and centrifuged at 13,000× *g* at room temperature for 10 min. The supernatant was filtered using a Sep-Pak^®^1cc (100 mg) C18 cartridge (Waters Corporation, Milford, MA, USA). Finally, an HPLC (high-performance liquid chromatography) system was employed to detect the sugars.

#### 2.5.2. Metabolite Detection

The sterilized samples were swiftly separated using a knife, with the middle of the pulp in both cases, JD and JW, being sliced into five pieces labeled JD1, JD2, JD3, JD4, and JD5 for JD, and YW1, YW2, YW3, YW4, and YW5 for YW. The sample information, sample grouping, and corresponding information are mentioned in Table S1. Then, 50 mg of lyophilized powder sample was dissolved in 0.6 mL of 70% methanol. Then, vortexes were performed once every 30 min, each lasting 30 s, and centrifuged at 12,000 rpm for 10 min. Finally, a microporous membrane (0.22 μm pore size) was used to filter the supernatant. The samples were analyzed by UPLC-ESI-MS/MS (Ultra-Performance Liquid Chromatography–Electrospray Tandem Mass Spectrometry) according to the method described by Chen et al. [45]. Qualitative and quantitative analyses of the metabolites were conducted using the self-built database MWDB (metware database; http://www.metware.cn/; accessed on 26 December 2023) [46].

### 2.6. In Silico Analysis

#### 2.6.1. Protein Preparation

The three-dimensional (3D) crystal structure of *E. coli* (PDB ID: 1HNJ), *Pseudomonas* sp. (PDB ID: 1U1Z), and *Staphylococcus* sp. (PDB ID: 1JIJ) proteins were retrieved from the Research Collaboratory for Structural Bioinformatics Protein Data Bank (RCSB PDB) [47]. With the latest PyMOL (Version 2.5.7) [48] and Discovery Studio [49] software (Version 24.1) packages. The extracted protein structure was cleaned and prepared by removing water molecules and heteroatoms. Following crystal structure purification, energy was optimized and minimized using the Swiss-PDB viewer [50] program with the Groningen Molecular Simulation (GROMOS) 43B1 force field. Minimizing the crystal conformation of the proteins involves optimizing side-chain geometry, missing hydrogen, and other inappropriate bonds.

#### 2.6.2. Ligand Preparation

Six hundred fifty-nine metabolites were detected in the dragon fruit pulp, including amino acids and derivatives, lipids, nucleotides and derivatives, organic acids, and various other metabolites. These metabolites were prepared as ligands in the molecular docking study. The steepest gradient approach and the mmff94 force field [51] minimized and optimized the ligand structures.

#### 2.6.3. Molecular Docking Study

Molecular docking was performed using the PyRx virtual screening program [52] to explore all possible configurations, positionings, and binding affinities for the ligands with the 1HNJ, 1U1Z, and 1JIJ proteins. All ligand-free energy values were optimized using the universal force field (UFF), conjugate gradient methods, and 2000 minimization steps. All ligands underwent conversion to PDBQT format to ensure docking in AutoDock Vina. Since the protein remains fixed, this docking technique was created to permit the rotation of any ligand. Table 2 includes each docked complex’s center and grid box dimensions. After docking, protein–ligand complexes were observed, docking poses were examined using BIOVIA Discovery Studio (Paris, France), and a non-bonding interaction study was conducted using PyMOL and BIOVIA Discovery Studio. A docking study was conducted to select the top two conformations for each protein based on their docking scores (in kcal/mol).

#### 2.6.4. Molecular Dynamics (MD) Simulation

With the aid of the AMBER14 force field [53,54], the YASARA dynamics software application [55,56] was implemented to execute the molecular dynamics (MD) simulation study. Initially, the docked complexes underwent cleaning, optimization, and hydrogen bond network alignment. With periodic boundary conditions, the TIP3P water solvation model was employed [57,58,59]. The simulations were performed under physiological circumstances, which included 298K, pH 7.4, and 0.9% NaCl. The steepest gradient tactics were employed with the simulated annealing technique (5000 cycles) to conduct initial energy minimizations with a time step of 1.25 fs. The Particle Mesh Ewald (PME) technique was implemented to calculate the long-range electrostatic interactions, and the cutoff radius was set to 8.0 Å [60,61,62,63,64]. Simulation trajectory data were saved every 100 ps. The simulations were kept going for 100 ns while adhering to the Berendsen thermostat, constant pressure, and temperature. The root mean square fluctuations (RMSFs), the radius of gyration (Rg), solvent accessible surface area (SASA), root mean square deviations (RMSDs), and hydrogen bonds were computed by employing the simulated trajectories [65,66,67].

#### 2.6.5. ADMET Prediction

The ADMET (absorption, distribution, metabolism, excretion, and toxicity) predictions obtained from reliable web tools like SwissADME [68], pKCSM [69], and admetSAR [70] were used to determine the pharmacokinetic properties. From the canonical SMILES (simplified molecular-input line-entry system) structures of the top two compounds for each protein, these web tools predicted features related to drug-likeness.

### 2.7. Statistical Analysis

The study had three replications for each biological sample [71]. The mean and standard deviation were calculated using Microsoft Excel version 2021 to determine the significance of the mean ± SD of each data group. GraphPad Prism 8 was used to generate graphical representations.

## 3. Results

### 3.1. Antibacterial Activity Test

**Figure 2:**
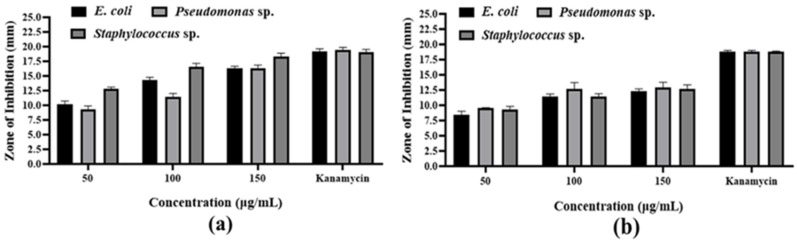
Antibacterial activity of fruit methanolic extracts of *S. undatus* varieties JD (**a**) and YW (**b**). Inhibition zone diameter values are expressed as mean ± SD.

The antibacterial activities of *S. undatus* varieties are demonstrated in Figure 2. The methanolic extract of the JD variety inhibited *E. coli*, *Pseudomonas* sp., and *Staphylococcus* sp. with a zone of 16.30 ± 0.36 mm, 16.37 ± 0.50 mm, and 18.33 ± 0.55 mm (Table S2, Figure 2), while the YW extract inhibited them with zones of 12.30 ± 0.40 mm, 12.90 ± 0.89 mm, and 12.63 ± 0.70 mm at the highest 150 μg/mL dose. According to these results, the JD variety exhibited higher levels of antibacterial activity against these three pathogenic bacteria than the YW variety.

### 3.2. Antioxidant Activity Test

**Figure 3:**
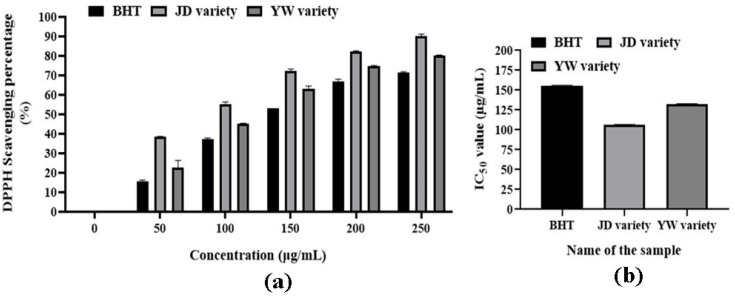
Antioxidant activities of *S. undatus* varieties. Here, (**a**) indicates the radical scavenging activities of JD and YW varieties, and (**b**) indicates the IC_50_ values of these varieties. The columns indicate significant differences between mean ± SD.

The antioxidant activities of *S. undatus* varieties were demonstrated by using the BHT standard, as illustrated in Figure 3 and Table S3. The scavenging percentage of these extracts increased gradually with the increase in the concentration of the extracts (Figure 3a). In contrast to the standard BHT (155.76 µg/mL), the IC_50_ values of JD and YW varieties were 106.14 µg/mL and 132.23 µg/mL, respectively. Here, the methanolic extract of the JD variety showed more significant antioxidant activity than the YW variety (Figure 3b), as it showed higher DPPH scavenging percentages at 50, 100, 150, 200, and 250 µg/mL concentrations, which was less in the YW variety.

### 3.3. Cytotoxic Activity Test

**Figure 4:**
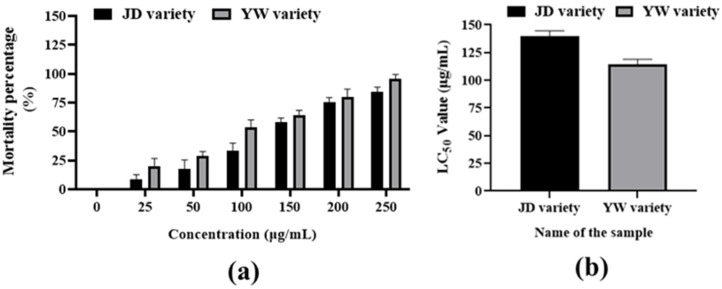
Cytotoxic activity of *S. undatus* dragon fruit varieties against brine shrimp. Here, (**a**) indicates the mortality percentages of both JD and YW varieties, and (**b**) indicates the LC_50_ values of both JD and YW varieties. The columns indicate significant differences between mean ± SD.

The cytotoxicity of these extracts in *Artemia salina* is shown in Figure 4 and Table S4. The results indicated that the LC_50_ values of JD and YW varieties were 139.77 and 113.78 µg/mL, respectively. These findings unequivocally demonstrated that the methanolic extract of the JD variety exhibited less cytotoxic activity than the YW variety (Figure 4a,b) because it revealed 50% mortality at 139.77 µg/mL concentration, which was higher than at other concentrations (25 µg/mL, 50 µg/mL, and 100 µg/mL).

### 3.4. Biochemical Analysis

#### 3.4.1. Sugar Content Analysis

**Figure 5:**
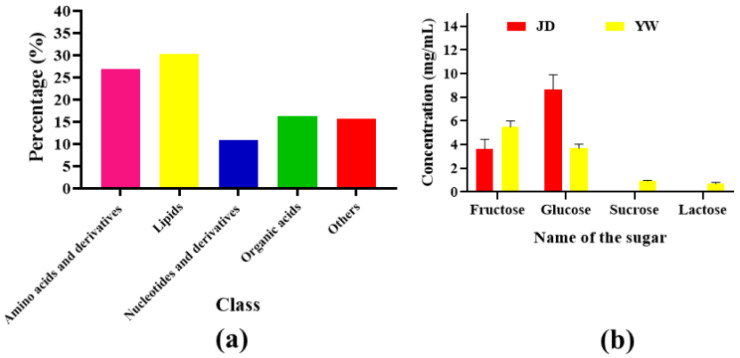
Metabolites classification and sugar concentration in two dragon varieties. Here, (**a**) indicates the percentages of the types of metabolite class, and (**b**) indicates differences in sugar concentrations between the JD and YW varieties. The error bars show statistically significant values between varieties.

The YW and JD varieties had a significant difference in sugar contents, as shown in Figure 5b. The fructose concentrations were 3.61 mg/mL and 5.49 mg/mL (Figure 5b and Table S5)**,** whereas the glucose concentrations were 8.66 mg/mL and 3.68 mg/mL in the JD and YW varieties, respectively (Figure 5b and Table S4). In addition, both sucrose and lactose were not present in the JD variety, but in the YW variety, were 0.93 mg/mL and 0.69 mg/mL, respectively.

#### 3.4.2. Qualitative Analysis of Metabolites

The total ion current (TIC) is shown in Figure S1a,b and the MRM metabolite detection multimodal diagram (XIC) is shown in Figure S1c,d. In metabolite detection, the perpendicular axis indicates the retention time (Rt), whereas the vertical axis indicates the ion detection intensity. The qualitative analyses of the metabolite samples were conducted using mass spectrometry and the local metabolic database. Each chromatographic peak with a distinct color denotes an identified metabolite. Each substance’s characteristic ions were removed using a triple quadrupole, and the detector’s signal intensity (CPS) was then measured in the sensor. After opening the mass spectrometry file of the sample using MultiQuant software (version 3.0.3), the peaks of the chromatograph were integrated and corrected. The relative amount of the equivalent substance was indicated by each chromatographic peak’s peak area (Area).

#### 3.4.3. Quantitative Analysis of Metabolites

A total of 659 metabolites were detected in the dragon fruit pulp, including amino acids and derivatives, lipids, nucleotides and derivatives, organic acids, and various other metabolites (Table S6, Table 1, and Figure 5a). Among the nine significant subclasses, 177 were amino acids and derivatives (26.86%), 199 were lipids (30.2%), followed by 119 free fatty acids, 61 lysophosphatidylcholines, 18 sphingolipids, and one phosphatidylcholine. The nucleotides and derivatives, organic acids, and other metabolites were 72 (10.93%), 108 (16.39%), and 103 (15.63%), respectively.

### 3.5. In Silico Analysis

#### 3.5.1. Molecular Docking Study

**Figure 6:**
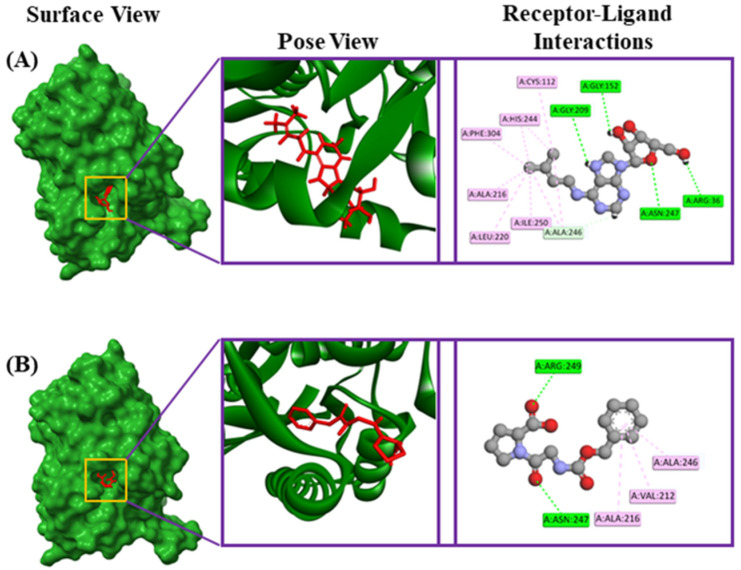
The various binding modes of riboprine (**A**) and Z-Gly-Pro (**B**) with the FabH protein of *E. coli* (PDB ID: 1HNJ).

**Figure 7:**
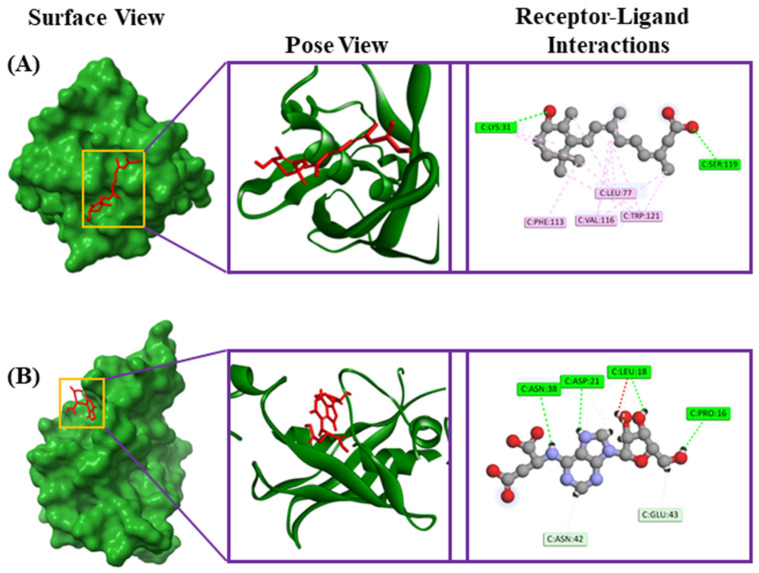
The various binding modes of 4-hydroxyretinoic acid (**A**) and succinyladenosine (**B**) with the FabZ protein of *Pseudomonas* sp. (PDB ID: 1U1Z).

**Figure 8:**
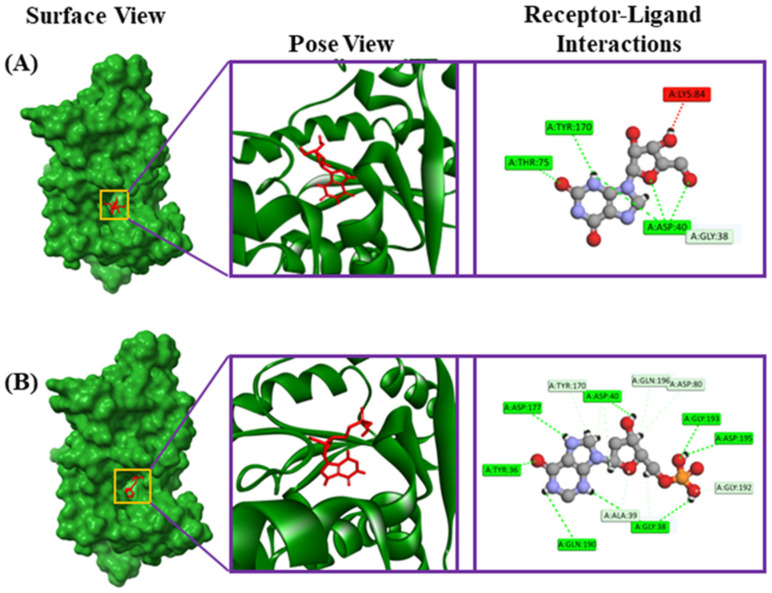
The various binding modes of xanthosine (**A**) and 2′-deoxyinosine-5′-monophosphate (**B**) with the tRNA syntheses proteins of *Staphylococcus* sp. (PDB ID: 1JIJ).

Riboprine and Z-Gly-Pro had binding affinities of −7.4 and −7.3 kcal/mol, respectively, with the FabH protein of *E. coli* (PDB ID: 1HNJ). 4-hydroxyretinoic acid and succinyladenosine had binding affinities of −6.8 and −6.6 kcal/mol, respectively, with the FabZ protein of *Pseudomonas* sp. (PDB ID: 1U1Z). Additionally, xanthosine and 2′-deoxyinosine-5′-monophosphate had binding affinities of −9.2 and −8.9 kcal/mol, respectively, with the tRNA syntheses proteins of *Staphylococcus* sp. (PDB ID: 1JIJ). The riboprine interacted with the 1HNJ through four conventional hydrogen bonds at Asn247, Arg36, Gly152, and Gly209 positions, one carbon–hydrogen bond at the Ala246 position, four alkyl bonds at Ala216, Leu220, Ile250, and Cys112 positions, and two pi-alkyl bonds at His244 and Phe304 positions (Table 3, Figure 6). The Z-Gly-Pro interacted with the 1HNJ through two conventional hydrogen bonds at Asn247 and Arg249 positions and three pi-alkyl bonds at Val212, Ala216, and Ala246 positions (Table 3, Figure 6). The 4-hydroxyretinoic acid–1U1Z complex formed two conventional hydrogen bonds at Lys31 and Ser119 positions, two alkyl bonds at Leu77 and Val116 positions, and two pi-alkyl bonds at Phe113 and Trp121 positions (Table 3, Figure 7). The succinyladenosine–1U1Z complex formed four conventional hydrogen bonds at Pro16, Leu18, Asn38, and Asp21 positions and two carbon hydrogen bonds at Asn42 and Glu43 positions (Table 3, Figure 7). Moreover, xanthosine was observed to form three conventional hydrogen bonds at the Asp40, Thr75, and Tyr170 positions, one carbon hydrogen bond at the Gly38 position, and one unfavorable donor–donor bond at the Lys84 position with 1JIJ (Table 3, Figure 8). In addition, 2′-deoxyinosine-5′-monophosphate was observed to form seven conventional hydrogen bonds at Tyr36, Gly193, Gln190, Asp195, Gly38, Asp40, and Asp177 positions and five carbon–hydrogen bonds at Ala39, Gly192, Asp80, Gln196, and Tyr170 positions (Table 3, Figure 8).

**Table 2:** The grid box and box size of the docked pose in AutoDock Vina.

Protein ID	Center (Å)	Dimension (Å)
1HNJ	X = 26.1977, Y = 25.9204, Z = 22.2037	X = 57.6841, Y = 56.5786, Z = 46.1378
1U1Z	X = 16.5014, Y = 8.3050, Z = 123.9474	X = 49.4324, Y = 35.9293, Z = 37.8294
1JIJ	X = 11.692, Y = 17.2899, Z = 91.7407	X = 43.9047, Y = 66.9841, Z = 51.4535

**Table 3:** Non-bond interactions of the ligand molecules with the three target proteins of *E. coli*, *Pseudomonas* sp., and *Staphylococcus* sp.

Complex	Binding Affinity (kcal/mol)	Residues in Contact	Interaction Type	Distance in Å
1HNJ–riboprine	−7.4	ASN247	Conventional Hydrogen Bond	2.48049
		ARG36	Conventional Hydrogen Bond	2.40409
		GLY152	Conventional Hydrogen Bond	2.40689
		GLY209	Conventional Hydrogen Bond	2.76002
		ALA246	Carbon–Hydrogen Bond	2.68055
		ALA216	Alkyl	4.13743
		LEU220	Alkyl	5.38211
		ILE250	Alkyl	4.67515
		CYS112	Alkyl	4.37411
		HIS244	Pi-Alkyl	5.11894
		PHE304	Pi-Alkyl	5.25225
1HNJ-Z-Gly-Pro	−7.3	ASN247	Conventional Hydrogen Bond	2.16212
		ARG249	Conventional Hydrogen Bond	2.71391
		VAL212	Pi-Alkyl	4.72913
		ALA216	Pi-Alkyl	5.22022
		ALA246	Pi-Alkyl	3.91873
1U1Z–4-Hydroxyretinoic acid	−6.8	LYS31	Conventional Hydrogen Bond	2.56031
		SER119	Conventional Hydrogen Bond	2.28802
		LEU77	Alkyl	4.26365
		VAL116	Alkyl	4.897
		PHE113	Pi-Alkyl	3.38907
		TRP121	Pi-Alkyl	4.56634
1U1Z–succinyladenosine	−6.6	PRO16	Conventional Hydrogen Bond	2.12334
		LEU18	Conventional Hydrogen Bond	2.08046
		ASN38	Conventional Hydrogen Bond	2.61959
		ASP21	Conventional Hydrogen Bond	2.48533
		ASN42	Carbon–Hydrogen Bond	2.26709
		GLU43	Carbon–Hydrogen Bond	2.20077
1JIJ–xanthosine	−9.2	ASP40	Conventional Hydrogen Bond	2.16622
		THR75	Conventional Hydrogen Bond	1.68242
		TYR170	Conventional Hydrogen Bond	2.35431
		GLY38	Carbon–Hydrogen Bond	3.02841
		LYS84	Unfavorable Donor–Donor Bond	1.58832
1JIJ–2′-deoxyinosine-5′-monophosphate	−8.9	TYR36	Conventional Hydrogen Bond	1.75377
		GLY193	Conventional Hydrogen Bond	2.20208
		GLN190	Conventional Hydrogen Bond	2.44649
		ASP195	Conventional Hydrogen Bond	2.33184
		GLY38	Conventional Hydrogen Bond	3.07379
		ASP40	Conventional Hydrogen Bond	2.99315
		ASP177	Conventional Hydrogen Bond	2.74948
		ALA39	Carbon–Hydrogen Bond	2.26262
		GLY192	Carbon–Hydrogen Bond	3.07554
		ASP80	Carbon–Hydrogen Bond	2.96818
		GLN196	Carbon–Hydrogen Bond	2.80142
		TYR170	Carbon–Hydrogen Bond	2.20065

#### 3.5.2. Molecular Dynamics (MD) Simulation

**Figure 9:**
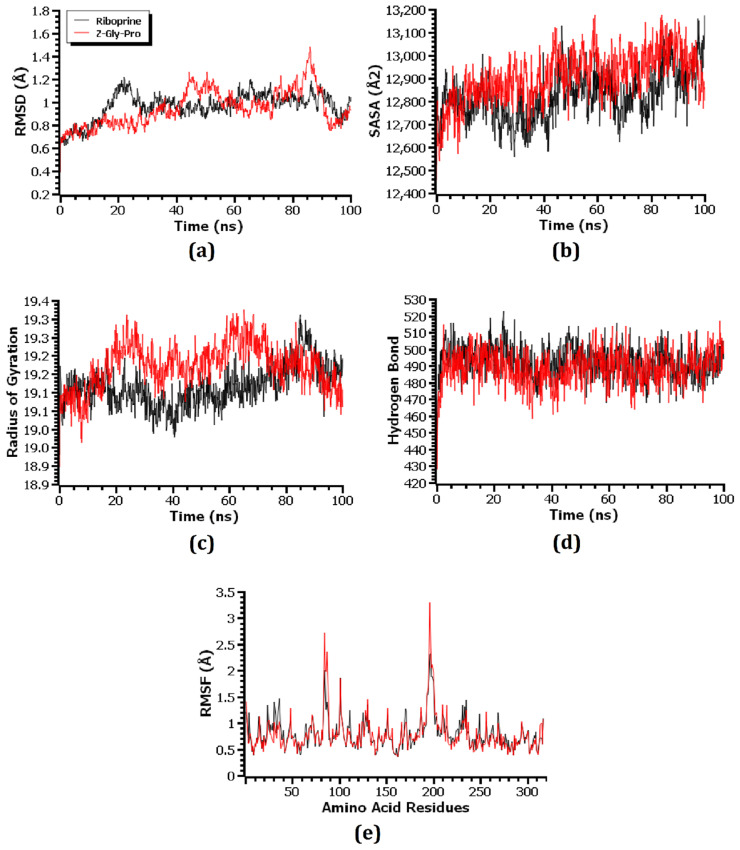
Molecular dynamics simulations of the compounds riboprine and Z-Gly-Pro in complex with 1HNJ protein. Here, (**a**–**e**) indicate (**a**) RMSD, (**b**) SASA, (**c**) Rg, (**d**) hydrogen bonds, and (**e**) RMSF analysis.

**Figure 10:**
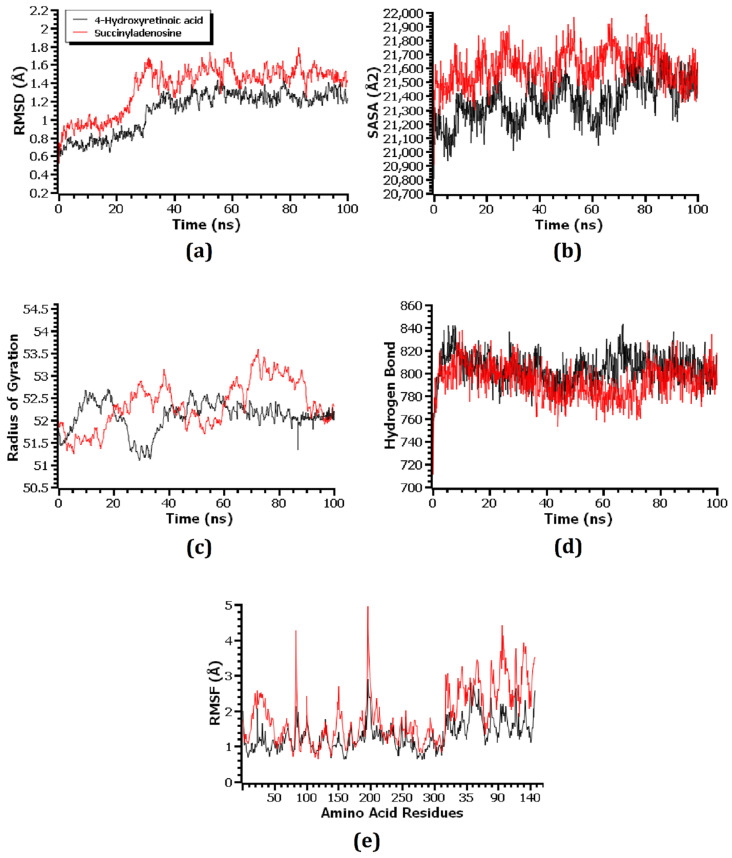
Molecular dynamics simulations of the compounds 4-hydroxyretinoic acid and succinyladenosine in complex with 1U1Z protein. Here, (**a**–**e**) indicate (**a**) RMSD, (**b**) SASA, (**c**) Rg, (**d**) hydrogen bonds, and (**e**) RMSF analysis.

**Figure 11:**
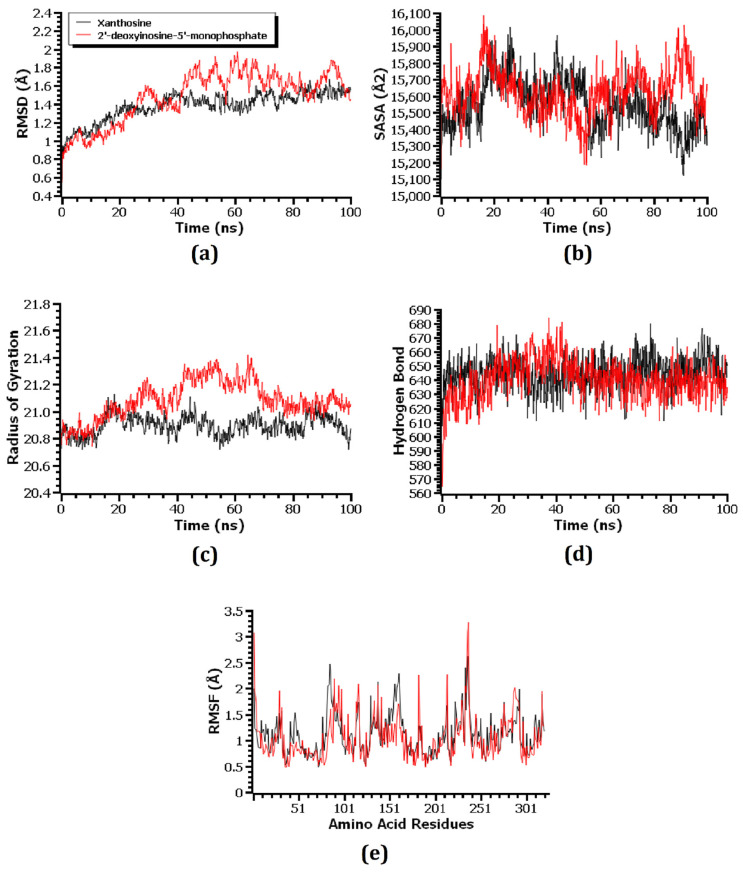
Molecular dynamics simulations of the compounds xanthosine and 2′-deoxyinosine-5′-monophosphate in complex with 1JIJ protein. Here, (**a**–**e**) indicate (**a**) RMSD, (**b**) SASA, (**c**) Rg, (**d**) hydrogen bonds, and (**e**) RMSF analysis.

Molecular dynamics (MD) simulation was used to investigate the structural stiffness of the 1HNJ–riboprine, 1HNJ-Z-Gly-Pro, 1U1Z–4-hydroxyretinoic acid, 1U1Z–succinyladenosine, 1JIJ–xanthosine, and 1JIJ–2′-deoxyinosine-5′-monophosphate complexes in order to validate the docking scenarios. The RMSD of the C-alpha atoms was calculated in order to understand the flexibility and stability status of the complex during the simulation period. A preliminary rise in the RMSD was seen in all six complexes, primarily mimicking their early flexibilities. The 1HNJ–riboprine and 1HNJ-Z-Gly-Pro complexes showed the highest rises in RMSD values after 20 ns and 85 ns of simulation, respectively (Figure 9a). The 1HNJ–riboprine complex stabilized at 28 ns and remained stable for the rest of the time, with minor fluctuation at the end of the simulation period. A few peaks were seen during the simulation, indicating that the 1HNJ-Z-Gly-Pro complex was less stable than the 1HNJ–riboprine complex. Both the 1U1Z–4-hydroxyretinoic acid and 1U1Z–succinyladenosine complexes stabilized after 30 ns of simulation and remained stable for the remaining time, with negligible fluctuations (Figure 10a). The 1U1Z–succinyladenosine complex had a higher RMSD value on average than the 1U1Z–4-hydroxyretinoic acid complex, indicating that the 1U1Z–4-hydroxyretinoic acid complex was more stable throughout the simulation. The 1JIJ–xanthosine complex became stable at around 20 ns and remained stable for the final 80 ns of simulation, with only minor variations (Figure 11a). Additionally, during the simulation period, the 1JIJ–2′-deoxyinosine-5′-monophosphate complex showed greater deviation, suggesting that it was less stable than the 1JIJ–xanthosine complex. However, during the 100 ns simulation period, all six complexes remained more or less stable because their RMSD values remained below 2.5 Å [63].

Since protein folding and inflexibility depend on this parameter, the SASA values were computed to investigate how docking with respective compounds affected the surface area of the 1HNJ, 1U1Z, and 1JIJ proteins [72]. In general, a protein with a higher SASA value has a larger surface area, while one with a low SASA value has a truncated structure [42]. Both the 1HNJ–riboprine and 1HNJ-Z-Gly-Pro complexes demonstrated an initial increase in the SASA value (Figure 9b). The SASA value of the 1HNJ-Z-Gly-Pro complex was greater on average during the simulation than the 1HNJ–riboprine complex, which indicated an increased surface area of the 1HNJ protein due to the interaction with the Z-Gly-Pro compound. Similarly, the 1U1Z–succinyladenosine and 1JIJ–2′-deoxyinosine-5′-monophosphate complexes had higher SASA values on average than the 1U1Z–4-hydroxyretinoic acid and 1JIJ–xanthosine complexes, which indicated enlargement of the surface area of the respective protein due to its interaction with the respective compound (Figure 10b and Figure 11b). Throughout the 100 ns simulation period, all six complexes exhibited more or less consistent SASA values, with the exception of a few minor variations.

Rg values were used to determine whether protein–ligand complexes were more flexible or rigid [54]. In the MD simulation, lower Rg values denoted more rigid protein–ligand complexes, whereas higher Rg values indicated more flexible protein–ligand complexes. The 1HNJ–riboprine complex remained stable throughout the 100 ns simulation period, with negligible deviations at 85 ns of simulation (Figure 9c). The Rg value of the 1HNJ–riboprine complex was lower on average than that of the 1HNJ-Z-Gly-Pro complex, which indicated that the 1HNJ–riboprine complex was stiffer. The Rg value of the 1U1Z–4-hydroxyretinoic acid complex stabilized at around 40 ns of the simulation and remained stable for the remaining 60 ns of the simulation (Figure 10c). Additionally, the 1U1Z–4-hydroxyretinoic acid complex had a lower Rg value on average than the 1U1Z–succinyladenosine complex, which indicated higher firmness of the 1U1Z–4-hydroxyretinoic acid complex. Additionally, both the 1JIJ–xanthosine and 1JIJ–2′-deoxyinosine-5′-monophosphate complexes had a steady Rg trend, with some minor ups and downs throughout the simulation, but the 1JIJ–xanthosine complex showed more inflexibility as it had a lower Rg value on average than the 1JIJ–2′-deoxyinosine-5′-monophosphate complex (Figure 11c).

The hydrogen bonds within the docked complexes were examined since they are essential to maintaining their integrity and rigidity during the simulation. All six complexes revealed a significant amount of hydrogen bonding throughout the 100 ns of MD simulation. In addition, the 1HNJ–riboprine, 1U1Z–4-hydroxyretinoic acid, and 1JIJ–xanthosine complexes displayed slightly more hydrogen bonding, which indicated more robust and inflexible complexes than the 1HNJ-Z-Gly-Pro, 1U1Z–succinyladenosine, and 1JIJ–2′-deoxyinosine-5′-monophosphate complexes (Figure 9d, Figure 10d and Figure 11d). In order to comprehend the flexibility of the 1HNJ, 1U1Z, and 1JIJ proteins across the amino acid residues, RMSF values of all the complexes were analyzed. The 1HNJ–riboprine, 1HNJ-Z-Gly-Pro, 1JIJ–xanthosine, and 1JIJ–2′-deoxyinosine-5′-monophosphate complexes had RMSF values less than 2.5 Å, with the exception of a few amino acid residues, whereas the 1U1Z–4-hydroxyretinoic acid and 1U1Z–succinyladenosine complexes had RMSF values less than 4 Å, except for a few amino acid residues (Figure 9e, Figure 10e and Figure 11e). Specifically, higher degrees of stiffness and inflexibility are associated with complexes with lower RMSF values [73] and the lower RMSF values of all six complexes implied that they remained stable throughout the 100 ns of the MD simulations.

#### 3.5.3. ADMET Prediction

Diverse ligand attributes, including pharmacokinetics and toxicity profiles, were assessed in order to determine the effectiveness and safety features of the top two candidate molecules for each protein (Table 4). Riboprine, Z-Gly-Pro, 4-hydroxyretinoic acid, succinyladenosine, xanthosine, and 2′-deoxyinosine-5′-monophosphate were found to have 7, 5, 3, 11, 7, and 9 hydrogen bond acceptors, and 4, 2, 2, 6, 5, and 4 hydrogen bond donors, respectively. The topological polar surface area (TPSA) was 125.55 Å^2^, 95.94 Å^2^, 57.53 Å^2^, 200.15 Å^2^, 153.46 Å^2^, and 169.60 Å^2^ for riboprine, Z-Gly-Pro, 4-hydroxyretinoic acid, succinyladenosine, xanthosine, and 2′-deoxyinosine-5′-monophosphate, respectively. Riboprine, Z-Gly-Pro, and 4-hydroxyretinoic acid exhibited high gastrointestinal (GI) absorption, whereas succinyladenosine, xanthosine, and 2′-deoxyinosine-5′-monophosphate showed low gastrointestinal (GI) absorption. All the top compounds, excluding 4-hydroxyretinoic acid, have been identified as being impermeable to the blood–brain barrier (BBB). Additionally, in toxicity profiling, none of the compounds exhibited skin sensitization. Moreover, all the compounds followed Lipinski’s rule of five, except succinyladenosine.

## 4. Discussion

As a source of innovative medication candidates and pharmacological treatments against infectious illnesses, phytochemicals produced by plants have recently attracted more attention [74]. Different varieties of dragon fruits may synthesize a wide range of metabolites with various biological activities that can be used by humans as a direct or indirect source of nourishment, energy, and medicine [3,75,76,77,78,79]. In this investigation, we scrutinized the metabolomic and biochemical profiles, as well as the cytotoxic and antioxidant properties of the Jindu No. 1 (red skin and red flesh) and Bird’s Nest (yellow skin and white flesh) varieties that are grown in China. Various in vitro and in silico investigations revealed that the *S. undatus* fruit may be useful as a natural source of drugs to control the three human pathogenic bacteria: *E. coli*, *Pseudomonas* sp., and *Staphylococcus* sp. Because of its wide range of applications in treating cancer, heart disease, vaginal discharge bleeding, hypertension, and type 2 diabetes, lowering harmful cholesterol levels, and creating new tissues, among others, it can be regarded as a good source of medicine [11,12,14,17].

In this study, the antibacterial activity test demonstrated that the methanolic extract of *S. undatus* varieties inhibited *E. coli*, *Pseudomonas* sp., and *Staphylococcus* sp., with the Jindu No. 1 (JD) fruit variety exhibiting higher antibacterial activity against these three pathogenic bacteria than the Bird’s Nest (YW) fruit variety. It is often a good indication that a plant extract’s antioxidant qualities may be able to block the enzymes required for microbial reproduction when phytoconstituents such as phenols, flavonoids, terpenoids, tannins, and steroids are present [80]. The best method for assessing the antioxidant capacity of plant components is to use a DPPH-free radical scavenging experiment [81]. The results of this investigation demonstrated that the two *S. undatus* varieties’ methanolic fruit extracts had higher antioxidant qualities; however, the JD fruit variety had greater antioxidant activity than the YW fruit variety. Additionally, several plants were shown to have a less harmful impact on brine shrimp in lethality testing [82]; this finding is strongly corroborated by the current research. The methanolic extract of the JD fruit variety showed better cytotoxic activity than that of the YW fruit variety on brine shrimp.

Previous research showed that fructose and glucose were the most prevalent sugars in pitaya. An investigation demonstrated that glucose is mostly present in pitaya, followed by fructose, while sucrose concentrations were comparatively low throughout fruit maturation [22,83,84,85,86]. According to another research, the sugars in dragon fruits were mostly fructose (28 to 40 mg/g) and glucose (60 to 65 mg/g), with smaller levels of sucrose (1.8 to 2.5 mg/g) making up around 2% of the fruit’s total sugar content [87]. Our research, however, yielded quite different findings about the sugar composition. In this present study, we found that the JD fruit variety had a higher amount of glucose, followed by fructose, whereas the YW fruit variety had a higher amount of fructose, followed by glucose, sucrose, and lactose.

In addition, the peel and pulp of green pitaya (*Hylocereus undatus*) and red pitaya (*Hylocereus polyrhizus*) were previously examined by Xing’e Lin et al. A total of 443 metabolites were identified in the peel and pulp of these two commercial pitaya varieties, including flavonoids, alkaloids, nucleotide and derivatives, amino acids and derivatives, coumarins, lipids, organic acids, lignans, phenolic acids, terpenoids, and others, through the UPLC-MS/MS technique [88]. Based on these results, pitaya contains large amounts of flavonoids, lipids, amino acids and derivatives, and phenolic acids. In another study, Yawei Wu et al. found a total of 65 metabolites, including betalains and betalain precursors, amino acids and amines, organic acids and fatty acids, sugars and sugar alcohols, and other compounds, in the *Hylocereus polyrhizus* fruit [89]. In the current study, 659 metabolites were detected in the dragon fruit pulp, including amino acids and derivatives, lipids, nucleotides and derivatives, organic acids, and various other metabolites. From these metabolites, lipids were present in higher amounts, followed by amino acids and derivatives, organic acids, nucleotides, and derivatives.

Beta-ketoacyl-acyl-carrier protein synthases, such as FabH, can start the production of fatty acids in bacteria and cause them to elongate [90]. Since the production of bacterial membranes depends on the fatty acid biosynthesis pathway, antibacterial medications find it to be a promising target. The protein FabZ in *Pseudomonas* sp. catalyzes this process of fatty acid production [91]. On the other hand, protein synthesis led by aminoacyl-tRNA synthetase can start the production of charged tRNA, which is a plausible target to combat the *Staphylococcus* sp. [92]. According to a previous study, the top two compounds against the FabH protein of *E. coli* displayed binding affinities of −7.6 and −8.1 kcal/mol. Moreover, the other top two compounds against the FabZ protein showed binding affinities of −8.4 and −7.9 kcal/mol. Furthermore, binding affinities of −7.9 and −8.7 kcal/mol were exhibited by other top two compounds against tRNA-synthetase [93]. In this investigation, riboprine and Z-Gly-Pro exhibited binding affinities of −7.4, and −7.3 kcal/mol, respectively with the FabH protein of *E. coli*. 4-hydroxyretinoic acid and succinyladenosine showed binding affinities of −6.8 and −6.6 kcal/mol, respectively, with the FabZ protein of *Pseudomonas* sp. Xanthosine and 2′-deoxyinosine-5′-monophosphate had binding affinities of −9.2 and −8.9 kcal/mol, respectively, with the tRNA syntheses proteins of *Staphylococcus* sp. All the selected compounds exhibited higher binding affinities in molecular docking analysis due to the formation of multiple non-bond interactions with the corresponding proteins of *E. coli*, *Pseudomonas* sp., and *Staphylococcus* sp. Additionally, all the selected compounds displayed acceptable ADMET properties to be plausible drug candidates against these three common pathogenic bacteria. In addition, all the docked protein–ligand complexes exhibited stability and firmness during the 100 ns of simulation.

In this investigation, we compared the sugar components of the JD and YW varieties that are grown in China and evaluated their antibacterial, antioxidant, and cytotoxic activities, and assessed their potential effects against *E. coli*, *Pseudomonas* sp., and *Staphylococcus* sp. The proportions of sugar components (fructose, glucose, sucrose, lactose) varied between the two varieties. Interestingly, our current research found that the JD fruit variety had a higher amount of glucose, followed by fructose, whereas the YW fruit variety had a higher amount of fructose, followed by glucose, sucrose, and lactose. The main reason that affects the differences in sugar components is the proportion of the different metabolites in the two varieties. The molecular docking program screened the top two candidates for each protein in *E. coli*, *Pseudomonas* sp., and *Staphylococcus* sp. This was further confirmed by 100 ns of MD simulations, and all these compounds displayed acceptable ADMET features. In short, the current study will provide vital molecular data to facilitate a deeper understanding of the two dragon fruit varieties and will considerably aid future investigations on this valuable fruit.

## 5. Conclusions

In this investigation, we examined the biochemical composition of the JD and YW varieties and assessed their antibacterial, antioxidant, and cytotoxic activities, as well as evaluated their potential effects against *E. coli*, *Pseudomonas* sp., and *Staphylococcus* sp. The methanolic extract of the JD variety showed higher antibacterial properties in vitro than the YW fruit variety against *E. coli*, *Pseudomonas* sp., and *Staphylococcus* sp. Both varieties had antioxidant and cytotoxic activities, but the JD variety had greater antioxidant and cytotoxic activities than the YW variety. Furthermore, this study is pioneering in reporting the variation in the proportion of sugar components (fructose, glucose, sucrose, lactose) between the two varieties. In the metabolomic analysis, 659 metabolites were found in the dragon fruit pulp, including amino acids and derivatives, lipids, nucleotides and derivatives, organic acids, and various other metabolites. Additionally, the molecular docking technique explored potential candidates against the three common pathogenic bacteria, including *E. coli*, *Pseudomonas* sp., and *Staphylococcus* sp., which were further confirmed by 100 ns of MD simulations, and all these compounds displayed acceptable ADMET features. Molecular data from this investigation will enable researchers to gain a deeper understanding of the two dragon fruit varieties and boost dragon fruit research in the future as a natural source of drugs for these three common pathogenic bacteria.

## Figures and Tables

**Table 1:** Classes and subclasses of metabolites detected in dragon fruit pulp.

Class	Count	Subclass	Count
Amino acids and derivatives	177	Amino acids and derivatives	177
Lipids	199	Free fatty acids	119
		Lysophosphatidylcholine	61
		Sphingolipids	18
		Phosphatidylcholines	1
Nucleotides and derivatives	72	Nucleotides and derivatives	72
Organic acids	108	Organic acids	108
Others	103	Saccharides	81
		Vitamins	22

**Table 4:** Pharmaceutical profiles derived from the SwissADME, admetSAR, and pKCSM webservers for the top two potential candidates for each protein obtained from docking.

Parameters	Riboprine	Z-Gly-Pro	4-Hydroxyretinoic Acid	Succinyladenosine	Xanthosine	2′-Deoxyinosine-5′-Monophosphate
Molecular Weight	335.36 g/mol	306.31 g/mol	316.43 g/mol	383.31 g/mol	284.23 g/mol	332.21 g/mol
Num. of H-bond acceptors	7	5	3	11	7	9
Num. of H-bond donors	4	2	2	6	5	4
TPSA (S)	125.55 Å^2^	95.94 Å^2^	57.53 Å^2^	200.15 Å^2^	153.46 Å^2^	169.60 Å^2^
GI absorption	High	High	High	Low	Low	Low
BBB permeant	No	No	Yes	No	No	No
Skin Sensitisation	No	No	No	No	No	No
Lipinski’s rule of five	Yes; 0 violation	Yes; 0 violation	Yes; 0 violation	No; 2 violations	Yes; 0 violation	Yes; 1 violation

## Data Availability

The original contributions presented in this study are included in the article and Supplementary Materials. Further inquiries can be directed to the corresponding author(s).

## Supplementary Materials

The following supporting information can be downloaded at: https://www.mdpi.com/article/10.3390/metabo14110577/s1, Figure S1: Mass spectrometry of mixed samples was used to analyze the total ion current plot. Here, (a) represents the negative ion mode, and (b) represents the positive ion mode, whereas (c) represents the multimodal pattern of MRM metabolite detection at the negative ion mode, and (d) represents the positive ion mode; Table S1: Sample grouping and sample number information of the dragon fruits used in the study. Here, JD indicates ‘Jindu No. 1 (red skin and red flesh)’ and YW indicates ‘bird’s nest (yellow skin and white flesh)’ dragon fruit varieties; Table S2: Inhibition zone of methanolic extract of *S. undatus* varieties with Kanamycin against selected bacterial strains; Table S3: DPPH scavenging activity of two dragon fruits in methanolic solvent extract with BHT standard; Table S4: Cytotoxic mortality percentage of two dragon fruit in methanolic solvent extract; Table S5: Sugar components concentration in the ‘Jindu No. 1 (JD)’ and ‘yellow skin and white flesh (YW)’ dragon fruit pulp, and Table S6: The total identified metabolites from the ‘Jindu No. 1 (JD) and Birds Nest (YW) dragon fruits varieties.
