# Supplementary material for: Selenicereus undatus (Dragon Fruit) Phytochemicals for Managing Three Human Pathogenic Bacteria: An In Vitro and In Silico Approach
Source: Metabolites. 2024 Oct 25;14(11):577. doi: 10.3390/metabo14110577 (PMC11596672; doi:10.3390/metabo14110577)
Supplement: Supplementary file 1 [file metabolites-14-00577-s001.zip › metabolites-3220725-supplementary.pdf]

Supplementary Materials

Selenicereus undatus (Dragon fruit) phytochemicals for managing three human pathogenic bacteria: An *in vitro* and *in silico* approach

Zhuan-Ying Yang, Xue-Wen Zheng, Wen-Hao Jiang, Gui-Zhi Chen, Qing-Zhi Liang, Guang-Zhao Xu * and Run-Hua Yi *

College of Coastal Agricultural Sciences, Guangdong Ocean University, Zhanjiang 524088, China; irene0411@gdou.edu.cn (Z.-Y.Y.); 2112104035@stu.gdou.edu.cn (X.-W.Z.); 2112204052@stu.gdou.edu.cn (W.-H.J.); 202111321102@stu.gdou.edu.cn (G.-Z.C.); qingzhi2002@gdou.edu.cn (Q.-Z.L.)

* Correspondence: xugz@gdou.edu.cn (G.-Z.X.); scibyrh@gdou.edu.cn (R.-H.Y.)

Supplementary Figures

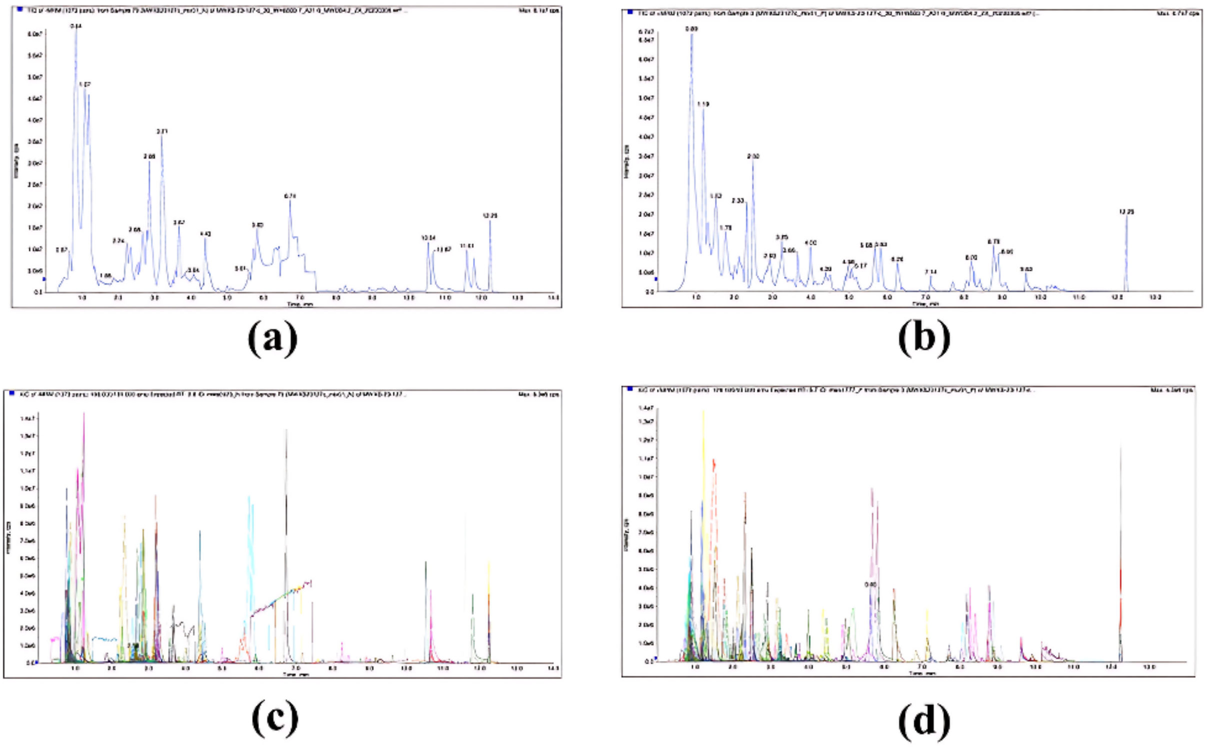

Figure S1. Mass spectrometry of mixed samples was used to analyze the total ion current plot. Here, **(a)** represents the negative ion mode, and **(b)** represents the positive ion mode, whereas **(c)** represents the multimodal pattern of MRM metabolite detection at the negative ion mode, and **(d)** represents the positive ion mode.

Supplementary Tables

Table S1. Sample grouping and sample number information of the dragon fruits used in the study. Here, JD indicates 'Jindu No. 1 (red skin and red flesh)' and YW indicates 'bird's nest (yellow skin and white flesh)' dragon fruit varieties.

Variety Name	Tissue Site	Sample Name	Group
Jindu No.1	Pulp	JD1-1	JD1
		JD1-2	JD1
		JD1-3	JD1
		JD2-1	JD2
		JD2-2	JD2
		JD2-3	JD2
		JD3-1	JD3
		JD3-2	JD3
		JD3-3	JD3
		JD4-1	JD4
		JD4-2	JD4
		JD4-3	JD4
		JD5-1	JD5
		JD5-2	JD5
		JD5-3	JD5
Bird's Nest	Pulp	YW1-1	YW1
		YW1-2	YW1
		YW1-3	YW1
		YW2-1	YW2
		YW2-2	YW2
		YW2-3	YW2
		YW3-1	YW3
		YW3-2	YW3
		YW3-3	YW3
		YW4-1	YW4
		YW4-2	YW4
		YW4-3	YW4
		YW5-1	YW5
		YW5-2	YW5
		YW5-3	YW5

Table S2. Inhibition zone of methanolic extract of *S. undatus* varieties with Kanamycin against selected bacterial strains.

Name of bacteria	Sample Dose (μg/mL)	Mean± SD Zone of Inhibition (mm)				Mean± SD of Kanamycin (30μg/dose)			
		Methanolic Extract of JD variety	S/I/R	Methanolic Extract of YW variety	S/I/R	JD variety	S/I/R	YW variety	S/I/R
E. coli	50	10.33±0.59	I	8.47±0.59	R	19.20±0.43	S	18.87±0.15	S
	100	14.33±0.47	I	11.40±0.46	I				
	150	16.30±0.36	S	12.30±0.40	I				
Pseudomonas sp.	50	9.37±0.51	I	9.53±0.06	R	19.46±0.42	S	18.80±0.20	S
	100	11.40±0.62	I	12.67±1.05	I				
	150	16.37±0.50	S	12.90±0.89	I				
Staphylococcus sp.	50	12.80±0.30	R	9.30±0.53	R	19.10±0.44	S	18.76±0.15	S
	100	16.57±0.59	S	11.43±0.47	I				
	150	18.33±0.55	S	12.63±0.70	I				

Note: Resistant: R indicates resistance, I indicates intermediate resistance, and S indicates susceptible, and R>10; 10-15= I; 15< S.

Table S3. DPPH scavenging activity of two dragon fruits in methanolic solvent extract with BHT standard.

Conc. (μg/mL)	Standard		Methanolic Extract			
	BHT Scavenging (%)	IC_{50} (μg/mL)	JD variety Scavenging (%)	IC_{50} (μg/mL)	YW variety Scavenging (%)	IC_{50} (μg/mL)
50	15.77±0.55	155.76	38.51±0.19	106.14	22.59±3.79	132.23
100	37.31±0.57		55.38±0.94		45.08±0.39	
150	53.29±0.55		72.24±1.01		63.04±1.52	
200	66.78±1.21		82.37±0.28		74.95±0.21	
250	71.35±0.56		90.31±0.87		80.41±0.05	

Table S4. Cytotoxic mortality percentage of two dragon fruit in methanolic solvent extract.

Conc. (μg/mL)	No. of Artemia taken	Methanolic extract			
		JD variety Mortality (%)	LC_{50} (μg/mL)	YW variety Mortality (%)	LC_{50} (μg/mL)
25	15	8.89±3.85	139.77	20.00±6.67	113.78
50		17.78±7.70		28.89±3.85	
100		33.33±6.66		53.33±6.67	
150		57.78±3.85		64.44±3.85	
200		75.56±3.85		80.00±6.67	
250		84.44±3.85		95.56±3.85	

Table S5. Sugar components concentration in the 'Jindu No. 1 (JD)' and 'yellow skin and white flesh (YW)' dragon fruit pulp.

Sugars	Samples	R1	R2	R3	Average	Final Concentration (mg/mL)	SD
Fructose	JD1	2.464	2.643	2.405	2.504	3.609	0.845
	JD2	4.178	3.885	3.987	4.017		
	JD3	4.552	4.45	4.428	4.477		
	JD4	4.207	4.235	3.906	4.116		
	JD5	3.013	2.951	2.831	2.932		
	YW1	4.668	4.558	4.825	4.684	5.485	0.517
	YW2	5.684	5.879	6.128	5.897		
	YW3	5.941	5.793	5.805	5.846		
	YW4	5.843	5.822	5.586	5.750		
	YW5	5.287	5.285	5.173	5.248		
Glucose	JD1	7.018	7.518	6.638	7.058	8.658	1.252
	JD2	9.191	9.181	8.976	9.116		
	JD3	10.204	10.062	9.995	10.087		
	JD4	9.02	10.297	8.753	9.357		
	JD5	7.657	7.607	7.753	7.672		
	YW1	3.142	2.84	3.292	3.091	3.677	0.357
	YW2	3.555	3.744	4.415	3.905		
	YW3	3.808	3.786	3.591	3.728		
	YW4	4.381	3.771	3.882	4.011		
	YW5	3.771	3.59	3.585	3.649		
Sucrose	JD1	0	0	0	0	0	0
	JD2	0	0	0	0		
	JD3	0	0	0	0		
	JD4	0	0	0	0		
	JD5	0	0	0	0		
	YW1	0.78	0.756	1.03	0.855	0.930	0.046
	YW2	1.041	0.902	0.875	0.939		
	YW3	0.955	1.005	0.959	0.973		
	YW4	0.849	1.091	0.942	0.961		
	YW5	0.873	1.055	0.824	0.917		
Lactose	JD1	0	0	0	0	0	0
	JD2	0	0	0	0		
	JD3	0	0	0	0		
	JD4	0	0	0	0		
	JD5	0	0	0	0		
	YW1	0.523	0.57	0.631	0.575	0.688	0.135
	YW2	0.742	0.807	0.985	0.845		
	YW3	0.713	0.88	0.834	0.809		
	YW4	0.745	0.551	0.71	0.669		
	YW5	20.068	0.635	0.718	0.544		

Table S6. The total identified metabolites from the 'Jindu No. 1 (JD) and Birds Nest (YW) dragon fruits varieties.

Sl No.	Compounds Name	Molecular weight (Da)	Formula	Class
1	5'-Deoxyadenosine*	2.51E+02	$C_{10}H_{13}N_5O_3$	Nucleotides and derivatives
2	Dimethylmalonic acid*	1.32E+02	$C_5H_8O_4$	Organic acids
3	2-Methylsuccinic acid*	1.32E+02	$C_5H_8O_4$	Organic acids
4	LysoPC 18:2(2n isomer)	5.19E+02	C26H50NO7P	Lipids
5	LysoPC 16:0(2n isomer)	4.95E+02	C24H50NO7P	Lipids
6	2-Aminotetradecane-1,4-diol	2.45E+02	C14H31NO2	Lipids
7	2-AminodocoSane-1,5,21-triol	3.73E+02	C22H47NO3	Lipids
8	1-(2,3-dihydroxypropoxy)-3-(((2-(dimethylamino)ethoxy)(hydroxy)phosphoryl)oxy)propan-2-yl (Z)-14-Octadecenoic Acid	5.81E+02	C28H56NO9P	Lipids
9	N-carboxy-N-(2-oxo-2-phenylethyl)-L-alanine	2.51E+02	C12H13NO5	Amino acids and derivatives
10	2-Hydroxy-4-methyl-3-undecanoyloxypentanoic acid methyl ester	3.30E+02	C18H34O5	Lipids
11	Sanleng acid	3.30E+02	C18H34O5	Lipids
12	LysoPC 16:0	4.95E+02	C24H50NO7P	Lipids
13	O-Acetyl-L-homoserine	1.61E+02	C6H11NO4	Amino acids and derivatives
14	LysoPC 18:1	5.21E+02	C26H52NO7P	Lipids
15	sorbose	1.80E+02	C6H12O6	Others
16	2-aminodocoSane-1,6,19,20,21-pentaol	4.05E+02	C22H47NO5	Lipids
17	D-Proline betaine	1.44E+02	C7H14NO2+	Amino acids and derivatives
18	LysoPC 18:2	5.19E+02	C26H50NO7P	Lipids
19	DL-Methionine	1.49E+02	C5H11NO2S	Amino acids and derivatives
20	14-Amino-15-hydroxy-11-methylpentadecanoic acid	2.87E+02	C16H33NO3	Lipids
21	Cys-Phe	2.68E+02	C12H16N2O3S1	Amino acids and derivatives
22	2-AminoicoSane-1,5,7,19-tetraol	3.61E+02	C20H43NO4	Lipids
23	Homoproline	1.29E+02	C6H11NO2	Amino acids and derivatives
24	3-(Beta-D-Glucopyranosyloxy)-5-Hydroxyhexanoic Acid Methyl Ester	3.24E+02	C13H24O9	Organic acids
25	LysoPC 18:1(2n isomer)	5.21E+02	C26H52NO7P	Lipids
26	N-(N-Formyl-L-methionyl)-L-phenylalanine	3.24E+02	C15H20N2O4S	Amino acids and derivatives
27	D-Erythronolactone	1.18E+02	C4H6O4	Organic acids
28	Succinyladenosine	3.83E+02	C14H17N5O8	Nucleotides and derivatives
29	Cycloleucine	1.29E+02	C6H11NO2	Amino acids and derivatives
30	2-[(1R,2R)-3-oxo-2-[(Z)-5-[3,4,5-trihydroxy-6-(hydroxymethyl)oxan-2-yl]oxypent-2-enyl]cyclopentyl]acetic acid	3.88E+02	C18H28O9	Lipids
31	9,10,11-Trihydroxy-12-octadecenoic acid	3.30E+02	C18H34O5	Lipids

32	2-AminoicoSane-1,5,19-triol	3.45E+02	C20H43NO3	Lipids
33	Hydroxy ricinoleic acid	3.14E+02	C18H34O4	Lipids
34	Tianshic acid	3.30E+02	C18H34O5	Organic acids
35	D-Threose	1.20E+02	C4H8O4	Others
36	DL-Tryptophan	2.04E+02	C11H12N2O2	Amino acids and derivatives
37	Pro-Asn	2.29E+02	C9H15N3O4	Amino acids and derivatives
38	alpha-Hydroxylinoleic acid*	2.96E+02	C18H32O3	Lipids
39	Oleamide (9-Octadecenamide)	2.81E+02	C18H35NO	Lipids
40	9,16-Dihydroxypalmitic acid	2.88E+02	C16H32O4	Lipids
41	2-Methylglutaric acid*	1.46E+02	C6H10O4	Organic acids
42	Azelaic acid	1.88E+02	C9H16O4	Organic acids
43	N-Methyl-L-proline	1.29E+02	C6H11NO2	Amino acids and derivatives
44	(2S)-2-amino-4-methyl-4-pentenoic acid	1.29E+02	C6H11NO2	Amino acids and derivatives
45	S-(Methyl)glutathione	3.21E+02	C11H19N3O6S	Amino acids and derivatives
46	L-Valinol	1.03E+02	C5H13NO	Amino acids and derivatives
47	LysoPC 18:3(2n isomer)	5.17E+02	C26H48NO7P	Lipids
48	2-Aminododecane-1,4-diol	2.17E+02	C12H27NO2	Lipids
49	Homovanillic acid sulfate	2.62E+02	C9H10O7S	Organic acids
50	2-Aminopurine	1.35E+02	C5H5N5	Nucleotides and derivatives
51	Punicic acid (9Z,11E,13Z-octadecatrienoic acid)	2.78E+02	C18H30O2	Lipids
52	LysoPC 18:3	5.17E+02	C26H48NO7P	Lipids
53	Nap-Tyr-OH	4.86E+02	C27H22N2O7	Amino acids and derivatives
54	6-O-methylguanine	1.65E+02	C6H7N5O	Nucleotides and derivatives
55	(2r,3s,4r,5s)-2-(6-aminopurin-9-yl)-5-(hydroxymethyl)oxolane-3,4-diol	2.67E+02	C10H13N5O4	Nucleotides and derivatives
56	L-Leucyl-L-phenylalanine	2.78E+02	C15H22N2O3	Amino acids and derivatives
57	Thr-TyrMe-OH	4.18E+02	C20H22N2O8	Amino acids and derivatives
58	L-Glutaminyl-L-tryptophan	3.32E+02	C16H20N4O4	Amino acids and derivatives
59	LysoPC 19:2	5.33E+02	C27H52NO7P	Lipids
60	12(13)Ep-9-KODE	3.10E+02	C18H30O4	Lipids
61	LysoPC 14:0	4.67E+02	C22H46NO7P	Lipids
62	2-aminodocoSane-1,5,7,21-tetraol	3.89E+02	C22H47NO4	Lipids
63	4-methyl-1,5,2,3-dioxadiazinan-2-amine	1.19E+02	C3H9N3O2	Nucleotides and derivatives
64	2-amino-4-hydroxy-3-((3,4,5-trihydroxytetrahydro-2H-pyran-2-yl)oxy)butanoic acid	2.67E+02	C9H17NO8	Amino acids and derivatives
65	Undecanedioic acid	2.16E+02	C11H20O4	Lipids
66	N-acetyl-tryptophan*	2.46E+02	C13H14N2O3	Amino acids and derivatives

67	LysoPE 15:0	4.39E+02	C20H42NO7P	Lipids
68	L-Isoleucyl-L-Aspartate	2.46E+02	C10H18N2O5	Amino acids and derivatives
69	LysoPE 16:1	4.51E+02	C21H42NO7P	Lipids
70	LysoPC 16:1(2n isomer)	4.93E+02	C24H48NO7P	Lipids
71	9,12,13-Trihydroxy-10,15-octadecadienoic acid	3.28E+02	C18H32O5	Lipids
72	Tridecanoic Acid	2.14E+02	C13H26O2	Lipids
73	1-Eicosanol	2.98E+02	C20H42O	Lipids
74	9-(Arabinosyl)hypoxanthine	2.68E+02	C10H12N4O5	Nucleotides and derivatives
75	linoleoyl ethanolamine	3.23E+02	C20H37NO2	Lipids
76	6-(((S)-1-carboxyethyl)amino)-4-hydroxyhexanoicacid	2.19E+02	C9H17NO5	Amino acids and derivatives
77	L-Cyclopentylglycine	1.43E+02	C7H13NO2	Amino acids and derivatives
78	(S)-4-amino-5-oxo-5-(pentylamino)pentanoic acid	2.16E+02	C10H20N2O3	Amino acids and derivatives
79	Ricinoleic acid	2.98E+02	C18H34O3	Lipids
80	2-Aminoisobutyric acid	1.03E+02	C4H9NO2	Organic acids
81	Gingerglycolipid B	6.78E+02	C33H58O14	Lipids
82	Tuberonic acid glucoside	3.88E+02	C18H28O9	Organic acids
83	Piperonylic acid	1.66E+02	C8H6O4	Organic acids
84	2-Aminohexadecane-1,5,6-triol	2.89E+02	C16H35NO3	Lipids
85	2-Aminohexadecane-1,16,16-triol	2.89E+02	C16H35NO3	Lipids
86	N-Acetyl-L-tyrosine	2.23E+02	C11H13NO4	Amino acids and derivatives
87	L-Norleucine*	1.31E+02	C6H13NO2	Amino acids and derivatives
88	14,15-Dehydrocrepenynic acid	2.76E+02	C18H28O2	Lipids
89	Glycerol 9(E),11(Z),13(E)-octadecatrienoyl ester	3.52E+02	C21H36O4	Lipids
90	Thr-HoPhe-OH	4.02E+02	C20H22N2O7	Amino acids and derivatives
91	LysoPC 16:1	4.93E+02	C24H48NO7P	Lipids
92	N6-(2-Hydroxyethyl)adenosine	3.11E+02	C12H17N5O5	Nucleotides and derivatives
93	5-Aminoimidazole ribonucleotide	2.95E+02	C8H14N3O7P	Nucleotides and derivatives
94	TyrMe-Leu-OH	4.30E+02	C22H26N2O7	Amino acids and derivatives
95	Met-Abu-OH	3.42E+02	C14H18N2O6S	Amino acids and derivatives
96	3-Hydroxyoctadecanoic Acid	3.00E+02	C18H36O3	Lipids
97	LysoPC 18:0	5.23E+02	C26H54NO7P	Lipids
98	cyclo-(Gly-Phe)	2.04E+02	C11H12N2O2	Amino acids and derivatives
99	Pyridoxine-5'-O-glucoside	3.31E+02	C14H21NO8	Others
100	N-Acetyl-L-leucine	1.73E+02	C8H15NO3	Amino acids and derivatives
101	LysoPC 19:2(2n isomer)	5.33E+02	C27H52NO7P	Lipids
102	2-α-Linolenoyl-glycerol*	3.52E+02	C21H36O4	Lipids
103	1-Monolinolenoyl-Rac-Glycerol	3.52E+02	C21H36O4	Lipids

104	Octadecanedioic acid	3.14E+02	C18H34O4	Lipids
105	LysoPE 18:2(2n isomer)	4.77E+02	C23H44NO7P	Lipids
106	1,14-Tetradecanedioic Acid	2.58E+02	C14H26O4	Lipids
107	Tridecanedioic acid	2.44E+02	C13H24O4	Lipids
108	4-Hydroxy-8-sphingenine	3.15E+02	C18H37NO3	Lipids
109	propyl-L-alanine	1.31E+02	C6H13NO2	Amino acids and derivatives
110	2-Aminohexadecane-1,5,15-triol	2.89E+02	C16H35NO3	Lipids
111	Nap-TyrMe-OH	5.00E+02	C28H24N2O7	Amino acids and derivatives
112	Gln-Asp	2.61E+02	C9H15N3O6	Amino acids and derivatives
113	4-O-beta-D-glucosyl-4-coumaric acid	3.26E+02	C15H18O8	Organic acids
114	1-Stearidonoyl-Glycerol	3.50E+02	C21H34O4	Lipids
115	2-Aminotetradecane-1,5,13-triol	2.61E+02	C14H31NO3	Lipids
116	LysoPC 18:0(2n isomer)	5.23E+02	C26H54NO7P	Lipids
117	LysoPE 14:0	4.25E+02	C19H40NO7P	Lipids
118	Octadeca-11E,13E,15Z-trienoic acid	2.78E+02	C18H30O2	Lipids
119	3-Hydroxy-palmitic acid methyl ester	2.86E+02	C17H34O3	Lipids
120	D-Allo-Isoleucine*	1.31E+02	C6H13NO2	Amino acids and derivatives
121	13-methylmyristic acid	2.42E+02	C15H30O2	Lipids
122	Aminomalonic acid	1.19E+02	C3H5NO4	Organic acids
123	LysoPC 15:0(2n isomer)	4.81E+02	C23H48NO7P	Lipids
124	2-Hydroxymyristic acid	2.44E+02	C14H28O3	Organic acids
125	Asp-Nap-OH	4.38E+02	C22H18N2O8	Amino acids and derivatives
126	Allitol	1.82E+02	C6H14O6	Others
127	2-Aminotetradecane-1,11,13-triol	2.61E+02	C14H31NO3	Lipids
128	LysoPE 18:0	4.81E+02	C23H48NO7P	Lipids
129	DL-O-tyrosine	1.81E+02	C9H11NO3	Amino acids and derivatives
130	TyrMe-Asp-OH	4.32E+02	C20H20N2O9	Amino acids and derivatives
131	N7-Methylguanosine	2.97E+02	C11H15N5O5	Nucleotides and derivatives
132	Cyclo(Phe-Glu)	2.76E+02	C14H16N2O4	Amino acids and derivatives
133	12-Hydroxyoctadecanoic acid	3.00E+02	C18H36O3	Lipids
134	LysoPC 19:1	5.35E+02	C27H54NO7P	Lipids
135	1-Octadecanol	2.70E+02	C18H38O	Lipids
136	Suberic Acid	1.74E+02	C8H14O4	Organic acids
137	L-γ-Glutamyl-L-leucine	2.60E+02	C11H20N2O5	Amino acids and derivatives
138	Glucopyranose 6-Hydroxydecanoate	3.50E+02	C16H30O8	Others
139	Leu-Asp	2.46E+02	C10H18N2O5	Amino acids and derivatives
140	LysoPE 16:0(2n isomer)	4.53E+02	C21H44NO7P	Lipids
141	Mandelic acid-β-glucoside	3.14E+02	C14H18O8	Organic acids
142	2-(Dimethylamino)guanosine	3.11E+02	C12H17N5O5	Nucleotides and derivatives

143	γ-Glutamyltyrosine	3.10E+02	C14H18N2O6	Amino acids and derivatives
144	9-Oxo-10,12-Octadecadienoic Acid	2.94E+02	C18H30O3	Lipids
145	Methyl 3-aminopropanoate	1.03E+02	C4H9NO2	Amino acids and derivatives
146	NG,NG-Dimethyl-L-arginine*	2.02E+02	C8H18N4O2	Amino acids and derivatives
147	13-Hydroperoxy-9Z,11E-octadecadienoic acid	3.12E+02	C18H32O4	Lipids
148	N-Acetyl-L-Tryptophan*	2.46E+02	C13H14N2O3	Amino acids and derivatives
149	L-Prolyl-L-Phenylalanine	2.62E+02	C14H18N2O3	Amino acids and derivatives
150	D-Maltose	3.42E+02	C12H22O11	Others
151	D-Lactose	3.42E+02	C12H22O11	Others
152	Isomaltulose	3.42E+02	C12H22O11	Others
153	Rabdosia acid A	2.94E+02	C18H30O3	Lipids
154	Monomethyl succinate	1.32E+02	C5H8O4	Organic acids
155	D-Pinitol	1.94E+02	C7H14O6	Others
156	DL-2-hydroxystearic acid*	3.00E+02	C18H36O3	Lipids
157	2,6-diamino-7-methyl-5-oxooctanoic acid	2.02E+02	C9H18N2O3	Amino acids and derivatives
158	N,N'-Dimethylarginine;SDMA*	2.02E+02	C8H18N4O2	Amino acids and derivatives
159	Woodorien	3.30E+02	C14H18O9	Others
160	Nystose	6.66E+02	C24H42O21	Others
161	S-(2-Carboxypropyl)cysteine	2.07E+02	C7H13NO4S	Amino acids and derivatives
162	1-Linoleoyl-2-Lysophosphatidic Acid Monobutylamine Ester	5.05E+02	C25H48NO7P	Lipids
163	1-Monomyristin	3.02E+02	C17H34O4	Lipids
164	Vidarabine	2.67E+02	C10H13N5O4	Nucleotides and derivatives
165	Phe-Thr	2.66E+02	C13H18N2O4	Amino acids and derivatives
166	Isonicotinic acid	1.23E+02	C6H5NO2	Others
167	Cordycepin (3'-Deoxyadenosine)*	2.51E+02	C10H13N5O3	Nucleotides and derivatives
168	17,18-DiHETE	3.36E+02	C20H32O4	Lipids
169	Arginine methyl ester	1.88E+02	C7H16N4O2	Amino acids and derivatives
170	LysoPE 18:3(2n isomer)	4.75E+02	C23H42NO7P	Lipids
171	11-Octadecanoic acid(Vaccenic acid)*	2.82E+02	C18H34O2	Lipids
172	1-α-Linolenoyl-glycerol*	3.52E+02	C21H36O4	Lipids
173	Isocytosine	1.11E+02	C4H5N3O	Nucleotides and derivatives
174	1-(2-Amino-4-methylpentanoyl)pyrrolidine-2-carboxylic acid	2.28E+02	C11H20N2O3	Amino acids and derivatives
175	N-Methyl-Trans-4-Hydroxy-L-Proline	1.45E+02	C6H11NO3	Amino acids and derivatives
176	1,6-anhydro-β-D-glucose	1.62E+02	C6H10O5	Others

177	Oxaceprol	1.73E+02	C7H11NO4	Amino acids and derivatives
178	D-Fructose	1.80E+02	C6H12O6	Others
179	(3-(carboxyamino)-2-methylpropanoyl)phenylalanine	2.94E+02	C14H18N2O5	Amino acids and derivatives
180	2'-O-Methyladenosine	2.81E+02	C11H15N5O4	Nucleotides and derivatives
181	5-Hydroxy-DL-tryptophan(5-HTP)	2.20E+02	C11H12N2O3	Amino acids and derivatives
182	Cetostearic acid	2.56E+02	C16H32O2	Lipids
183	1-Linoleoylglycerol-2,3-di-O-glucoside*	6.78E+02	C33H58O14	Lipids
184	Citric acid glucoside	3.54E+02	C12H18O12	Organic acids
185	LysoPC 20:1	5.49E+02	C28H56NO7P	Lipids
186	Tyr-Gly	2.38E+02	C11H14N2O4	Amino acids and derivatives
187	1-Methyladenine	1.49E+02	C6H7N5	Nucleotides and derivatives
188	1-(2,3-dihydroxypropoxy)-3-(((2-(dimethylamino)ethoxy)(hydroxy)phosphoryl)oxy)propan-2-yl (11Z,14Z)-octadeca-11,14-dienoate*	5.79E+02	C28H54NO9P	Lipids
189	N-(beta-D-Glucosyl)nicotinate	2.85E+02	C12H15NO7	Others
190	LysoPE 16:0	4.53E+02	C21H44NO7P	Lipids
191	LysoPC 12:0	4.39E+02	C20H42NO7P	Lipids
192	2-amino-3-(1H-pyrazol-1-yl)propanoic acid	1.55E+02	C6H9N3O2	Organic acids
193	4-Oxatetradecanoic acid	2.30E+02	C13H26O3	Organic acids
194	L-Fucitol	1.66E+02	C6H14O5	Others
195	Glu-Phe	2.94E+02	C14H18N2O5	Amino acids and derivatives
196	Tyr-HoPhe-OH	4.64E+02	C25H24N2O7	Amino acids and derivatives
197	1-(2,3-dihydroxypropoxy)-3-(((2-(dimethylamino)ethoxy)(hydroxy)phosphoryl)oxy)propan-2-yl palmitate*	5.55E+02	C26H54NO9P	Lipids
198	Cytarabine	2.43E+02	C9H13N3O5	Nucleotides and derivatives
199	2R-Hydroxyoctadecanoic Acid*	3.00E+02	C18H36O3	Lipids
200	LysoPC 16:2	4.91E+02	C24H46NO7P	Lipids
201	Methyl palmitate	2.70E+02	C17H34O2	Lipids
202	LysoPE 18:3	4.75E+02	C23H42NO7P	Lipids
203	1-Oleoyl-Sn-Glycerol	3.56E+02	C21H40O4	Lipids
204	Hydroperoxylinoleic acid	3.12E+02	C18H32O4	Lipids
205	3-Hydroxyglutaric acid	1.48E+02	C5H8O5	Organic acids
206	L-α-Glutamyl-L-Glutamic Acid	2.76E+02	C10H16N2O7	Amino acids and derivatives
207	Monolinolenin	3.52E+02	C21H36O4	Lipids
208	LysoPC 20:2(2n isomer)	5.47E+02	C28H54NO7P	Lipids
209	Tyr-Nap-OH	5.00E+02	C28H24N2O7	Amino acids and derivatives
210	Prolylproline	2.12E+02	C10H16N2O3	Amino acids and derivatives
211	LysoPC 18:4	5.15E+02	C26H46NO7P	Lipids

212	4-Pyridoxic acid-O-glucoside	3.45E+02	C14H19NO9	Others
213	Glucosyl 2-Hydroxy-4-Methylpentanoic Acid	2.94E+02	C12H22O8	Organic acids
214	L-Glycyl-L-proline	1.72E+02	C7H12N2O3	Amino acids and derivatives
215	9s,13r-12-Oxophytodienoic Acid	2.92E+02	C18H28O3	Lipids
216	Asp-Ile-Leu	3.59E+02	C16H29N3O6	Amino acids and derivatives
217	Methyl linolenate	2.92E+02	C19H32O2	Lipids
218	LysoPC 17:1	5.07E+02	C25H50NO7P	Lipids
219	Uric acid	1.68E+02	C5H4N4O3	Nucleotides and derivatives
220	Cyclocreatine	1.43E+02	C5H9N3O2	Nucleotides and derivatives
221	Pyroglutamic acid	1.29E+02	C5H7NO3	Amino acids and derivatives
222	LysoPE 18:2	4.77E+02	C23H44NO7P	Lipids
223	3-Hydroxy-L-phenylalanine	1.81E+02	C9H11NO3	Amino acids and derivatives
224	Homoarginine	1.88E+02	C7H16N4O2	Amino acids and derivatives
225	9-Hydroxy-13-oxo-10-octadecenoic Acid	3.12E+02	C18H32O4	Lipids
226	TyrMe-Met-OH	4.48E+02	C21H24N2O7S	Amino acids and derivatives
227	Mandelic acid	1.52E+02	C8H8O3	Organic acids
228	9-Oxo-12Z-Octadecenoic acid	2.96E+02	C18H32O3	Lipids
229	Ribosyladenosine	3.99E+02	C15H21N5O8	Nucleotides and derivatives
230	LysoPC 15:0	4.81E+02	C23H48NO7P	Lipids
231	3-Oxopentanedioic acid	1.46E+02	C5H6O5	Organic acids
232	N-Monomethyl-L-arginine	1.88E+02	C7H16N4O2	Amino acids and derivatives
233	Methyl dihydrojasmonate	2.26E+02	C13H22O3	Organic acids
234	5-methyl 1-propyl L-glutamate	2.03E+02	C9H17NO4	Amino acids and derivatives
235	2-(2,3-dihydroxypropoxy)-3-(((2-(dimethylamino)ethoxy)(hydroxy)phosphoryl)oxy)propan-2-yl (Z)-14-Octadecenoic Acid	5.81E+02	C28H56NO9P	Lipids
236	2-(2,3-dihydroxypropoxy)-3-(((2-(dimethylamino)ethoxy)(hydroxy)phosphoryl)oxy)propyl (11Z,14Z)-octadeca-11,14-dienoate*	5.79E+02	C28H54NO9P	Lipids
237	Ile-Phe	2.78E+02	C15H22N2O3	Amino acids and derivatives
238	2-Aminooctadecane-1,5,7,17-tetraol	3.33E+02	C18H39NO4	Lipids
239	N-Glycyl-L-leucine*	1.88E+02	C8H16N2O3	Amino acids and derivatives
240	Iminodiacetic acid	1.33E+02	C4H7NO4	Organic acids
241	D-Cellobiose	3.42E+02	C12H22O11	Others
242	L-Alanyl-L-Phenylalanine	2.36E+02	C12H16N2O3	Amino acids and derivatives
243	Allantoin	1.58E+02	C4H6N4O3	Organic acids

244	3-(triazan-2-yl)propyl L-prolinate	2.02E+02	C8H18N4O2	Amino acids and derivatives
245	Glycylphenylalanine*	2.22E+02	C11H14N2O3	Amino acids and derivatives
246	Undecylic Acid	1.86E+02	C11H22O2	Lipids
247	L-Aspartic acid-O-diglucoside	4.57E+02	C16H27NO14	Amino acids and derivatives
248	TyrMe-His-OH	4.54E+02	C22H22N4O7	Amino acids and derivatives
249	L-Prolyl-L-Leucine	2.28E+02	C11H20N2O3	Amino acids and derivatives
250	L-Xylose	1.50E+02	C5H10O5	Others
251	LysoPE 17:1(2n isomer)	4.65E+02	C22H44NO7P	Lipids
252	L-Valyl-L-Phenylalanine	2.64E+02	C14H20N2O3	Amino acids and derivatives
253	γ-Glutamyl-L-valine	2.46E+02	C10H18N2O5	Amino acids and derivatives
254	6-Hydroxyhexanoic acid	1.32E+02	C6H12O3	Organic acids
255	Gingerglycolipid A	6.76E+02	C33H56O14	Lipids
256	1-Palmitoyl-Sn-Glycerol 3-O-Diglucoside	6.54E+02	C31H58O14	Lipids
257	Val-Val	2.16E+02	C10H20N2O3	Amino acids and derivatives
258	(2r,3s,4s,5r)-2,5-bis(hydroxymethyl)-2-methoxyoxolane-3,4-diol	1.94E+02	C7H14O6	Others
259	Phe-Ser	2.52E+02	C12H16N2O4	Amino acids and derivatives
260	Phe-Abu-OH	3.58E+02	C18H18N2O6	Amino acids and derivatives
261	LysoPC 20:2	5.47E+02	C28H54NO7P	Lipids
262	Ile-Abu-OH	3.24E+02	C15H20N2O6	Amino acids and derivatives
263	Crotonoside; 2-Hydroxyadenosine	2.83E+02	C10H13N5O5	Nucleotides and derivatives
264	Hydroxypentadecenoic acid glucoside	4.18E+02	C21H38O8	Lipids
265	Tyrosylleucine	2.94E+02	C15H22N2O4	Amino acids and derivatives
266	15(R)-Hydroxylinoleic Acid	2.96E+02	C18H32O3	Lipids
267	2-O-α-D-Glucopyranosyl-L-ascorbic acid	3.38E+02	C12H18O11	Others
268	1-O-Linoleoyl-3-O-galactopyranosyl-L-glycerol	5.16E+02	C27H48O9	Lipids
269	N-methylphenylalanine	1.79E+02	C10H13NO2	Amino acids and derivatives
270	Heptadecanoic acid	2.70E+02	C17H34O2	Lipids
271	LysoPC 17:2	5.05E+02	C25H48NO7P	Lipids
272	1-(2,3-dihydroxypropoxy)-3-(((2-(dimethylamino)ethoxy)(hydroxy)phosphoryl)oxy)propan-2-yl (8E,11Z,14Z)-octadeca-8,11,14-trienoate*	5.77E+02	C28H52NO9P	Lipids
273	Glycyl-tryptophan	2.61E+02	C13H15N3O3	Amino acids and derivatives
274	9,12-Octadecadien-6-Ynoic Acid	2.76E+02	C18H28O2	Lipids
275	LysoPC 17:0	5.09E+02	C25H52NO7P	Lipids
276	Dodecanedioic aicd	2.30E+02	C12H22O4	Lipids

277	LysoPE 20:4	5.01E+02	C25H44NO7P	Lipids
278	6-Hydroxydopaquinone	2.11E+02	C9H9NO5	Amino acids and derivatives
279	LysoPC 15:1	4.79E+02	C23H46NO7P	Lipids
280	LysoPE 18:0(2n isomer)	4.81E+02	C23H48NO7P	Lipids
281	Parinaraldehyde	2.60E+02	C18H28O	Lipids
282	N(6),N(6)-Dimethyl-L-lysine	1.74E+02	C8H18N2O2	Amino acids and derivatives
283	2-α-Linolenoyl-glycerol-1,3-di-O-glucoside*	6.76E+02	C33H56O14	Lipids
284	Petroselinic acid*	2.82E+02	C18H34O2	Lipids
285	Elaidic Acid*	2.82E+02	C18H34O2	Lipids
286	1,5-Anhydro-D-glucitol	1.64E+02	C6H12O5	Others
287	LysoPC(18:3(9Z,12Z,15Z))	5.17E+02	C26H48NO7P	Lipids
288	LysoPE 20:4(2n isomer)	5.01E+02	C25H44NO7P	Lipids
289	6-O-alpha-L-arabinopyranosyl-D-glucopyranose	3.12E+02	C11H20O10	Others
290	Gingerglycolipid C	6.80E+02	C33H60O14	Lipids
291	2,3-Dihydroxy-3-Methylbutanoic Acid	1.34E+02	C5H10O4	Organic acids
292	7-Methylguanine	1.65E+02	C6H7N5O	Nucleotides and derivatives
293	Maltitol	3.44E+02	C12H24O11	Others
294	LysoPE 15:0(2n isomer)	4.39E+02	C20H42NO7P	Lipids
295	1-beta-D-Arabinofuranosyluracil	2.44E+02	C9H12N2O6	Nucleotides and derivatives
296	2-Linoleoylglycerol-1-O-glucoside*	5.16E+02	C27H48O9	Lipids
297	LysoPE 18:1	4.79E+02	C23H46NO7P	Lipids
298	Isoguanine	1.51E+02	C5H5N5O	Nucleotides and derivatives
299	1-(2,3-dihydroxypropoxy)-3-(((2-(dimethylamino)ethoxy)(hydroxy)phosphoryl)oxy)propan-2-yl (E)-hexadec-9-enoate	5.53E+02	C26H52NO9P	Lipids
300	(6R)-5-Methyltetrahydrofolic acid	4.59E+02	C20H25N7O6	Organic acids
301	LysoPE 18:1(2n isomer)	4.79E+02	C23H46NO7P	Lipids
302	5,6-Dihydro-5-methyluracil	1.28E+02	C5H8N2O2	Nucleotides and derivatives
303	Uridine-5'-diphosphoglucuronic acid	5.80E+02	C15H22N2O18 P2	Nucleotides and derivatives
304	2-(2,3-dihydroxypropoxy)-3-(((2-(dimethylamino)ethoxy)(hydroxy)phosphoryl)oxy)propyl palmitate*	5.55E+02	C26H54NO9P	Lipids
305	Phe-Ala-Phe	3.83E+02	C21H25N3O4	Amino acids and derivatives
306	1-Linoleoylglycerol*	3.54E+02	C21H38O4	Lipids
307	S-Adenosylmethionine	3.99E+02	C15H23N6O5S +	Amino acids and derivatives
308	L-Glycyl-L-isoleucine*	1.88E+02	C8H16N2O3	Amino acids and derivatives
309	2-Methyl-4-pentenoic Acid	1.14E+02	C6H10O2	Organic acids
310	5-{[3,4-dihydroxy-2,5-bis(hydroxymethyl)oxolan-2-yl]oxy}-2-(hydroxymethyl)oxane-2,3,4-triol	3.42E+02	C12H22O11	Others
311	Diethyl phosphate	1.54E+02	C4H11O4P	Organic acids

312	Nap-Asp-OH	4.38E+02	C22H18N2O8	Amino acids and derivatives
313	3-Furoic acid	1.12E+02	C5H4O3	Organic acids
314	Isopentenyladenine-7-N-glucoside	3.65E+02	C16H23N5O5	Nucleotides and derivatives
315	2-Hydroxyisocaproic acid	1.32E+02	C6H12O3	Organic acids
316	Phylloquinone (Vitamin K1)	4.50E+02	C31H46O2	Others
317	L-Valyl-L-Leucine	2.30E+02	C11H22N2O3	Amino acids and derivatives
318	6-Benzylaminopurine	2.25E+02	C12H11N5	Nucleotides and derivatives
319	Tranexamic Acid	1.57E+02	C8H15NO2	Organic acids
320	Val-Pro	2.14E+02	C10H18N2O3	Amino acids and derivatives
321	1-Linoleoylglycerol-3-O-glucoside*	5.16E+02	C27H48O9	Lipids
322	N-Acetyl-L-Glutamine	1.88E+02	C7H12N2O4	Amino acids and derivatives
323	N-Alpha-Acetyl-L-Asparagine	1.74E+02	C6H10N2O4	Amino acids and derivatives
324	2-Linoleoylglycerol-1,3-di-O-glucoside*	6.78E+02	C33H58O14	Lipids
325	γ-Glutamylphenylalanine	2.94E+02	C14H18N2O5	Amino acids and derivatives
326	3-Hydroxy-3-methylpentane-1,5-dioic acid	1.62E+02	C6H10O5	Amino acids and derivatives
327	D-Melezitose	5.04E+02	C18H32O16	Others
328	Pro-Trp	3.01E+02	C16H19N3O3	Amino acids and derivatives
329	His-Nap-OH	4.74E+02	C25H22N4O6	Amino acids and derivatives
330	2-Linoleoylglycerol*	3.54E+02	C21H38O4	Lipids
331	Abu-Met-OH	3.42E+02	C14H18N2O6S	Amino acids and derivatives
332	N-(p-hydroxybenzyl) adenosine	4.03E+02	C18H21N5O6	Nucleotides and derivatives
333	Ethyl linoleate	3.08E+02	C20H36O2	Lipids
334	1-O-Acetyl-Glucopyranose 6-Hydroxydecanoate	3.92E+02	C18H32O9	Others
335	Monopalmitin	3.30E+02	C19H38O4	Lipids
336	D-Panose	5.04E+02	C18H32O16	Others
337	L-Glutamic acid-O-glycoside	3.09E+02	C11H19NO9	Amino acids and derivatives
338	Palmitoylethanolamide	2.99E+02	C18H37NO2	Lipids
339	Citric acid-1-O-diglucoside	5.16E+02	C18H28O17	Organic acids
340	LysoPC 16:2(2n isomer)	4.91E+02	C24H46NO7P	Lipids
341	L-Tyrosine methyl ester	1.95E+02	C10H13NO3	Amino acids and derivatives
342	LysoPE 16:1(2n isomer)	4.51E+02	C21H42NO7P	Lipids
343	LysoPE 17:1	4.65E+02	C22H44NO7P	Lipids
344	Bestim	3.33E+02	C16H19N3O5	Amino acids and derivatives
345	LysoPE 20:2	5.05E+02	C25H48NO7P	Lipids

346	Tyr-Met-OH	4.34E+02	C20H22N2O7S	Amino acids and derivatives
347	β-Crocetin	3.42E+02	C21H26O4	Organic acids
348	1-α-Linolenoyl-glycerol-2,3-di-O-glucoside*	6.76E+02	C33H56O14	Lipids
349	N-(3,4,5-trihydroxycinnamicacid)-alanine	2.67E+02	C12H13NO6	Amino acids and derivatives
350	L-Glycyl-L-phenylalanine*	2.22E+02	C11H14N2O3	Amino acids and derivatives
351	Ile-Trp	3.17E+02	C17H23N3O3	Amino acids and derivatives
352	Phe-Ile	2.78E+02	C15H22N2O3	Amino acids and derivatives
353	2-(2,3-dihydroxypropoxy)-3-(((2-(dimethylamino)ethoxy)(hydroxy)phosphoryl)oxy)propyl (8E,11Z,14Z)-octadeca-8,11,14-trienoate*	5.77E+02	C28H52NO9P	Lipids
354	L-Glutamine-O-glycoside	3.08E+02	C11H20N2O8	Amino acids and derivatives
355	1-(sn-Glycero-3-phospho)-1D-myo-inositol	3.34E+02	C9H19O11P	Others
356	Dambonitol	2.08E+02	C8H16O6	Others
357	(Oxiran-2-yl)methyl octadeca-9,12-dienoate	3.36E+02	C21H36O3	Lipids
358	N-Acetyl-L-threonine	1.61E+02	C6H11NO4	Amino acids and derivatives
359	5-Methyl-2'-Deoxycytidine	2.41E+02	C10H15N3O4	Nucleotides and derivatives
360	Sucrose-6-phosphate	4.22E+02	C12H23O14P	Others
361	Glycidyl Linoleate	3.36E+02	C21H36O3	Lipids
362	N5-(1-Iminoethyl)-L-ornithine	1.73E+02	C7H15N3O2	Amino acids and derivatives
363	(R)-Beta-Hydroxypalmitic Acid	2.72E+02	C16H32O3	Lipids
364	LysoPE 20:2(2n isomer)	5.05E+02	C25H48NO7P	Lipids
365	10-Hydroxystearic Acid	3.00E+02	C18H36O3	Lipids
366	L-Seryl-L-Isoleucine	2.18E+02	C9H18N2O4	Amino acids and derivatives
367	N-Propionylglycine	1.31E+02	C5H9NO3	Amino acids and derivatives
368	Glycyl-L-leucine	1.88E+02	C8H16N2O3	Amino acids and derivatives
369	N-acetyl-leucine	1.73E+02	C8H15NO3	Amino acids and derivatives
370	2-Palmitoyl-Sn-Glycerol 3-O-Diglucoside	6.54E+02	C31H58O14	Lipids
371	Glucan	5.04E+02	C18H32O16	Others
372	16-Methylheptadecanoic acid	2.84E+02	C18H36O2	Lipids
373	D-Maltotetraose	6.66E+02	C24H42O21	Others
374	L-Leucyl-L-Leucine	2.44E+02	C12H24N2O3	Amino acids and derivatives
375	1-Linoleoyl-2-Lysophosphatidic Acid Monomethyl Ester	4.48E+02	C22H41O7P	Lipids
376	Sebacic acid	2.02E+02	C10H18O4	Organic acids
377	PS(18:3(6Z,9Z,12Z)/0:0)	5.19E+02	C24H42NO9P	Lipids
378	Glucaric acid-1-Phosphate	2.90E+02	C6H11PO11	Others
379	2-Hydroxyhexadecanoic acid	2.72E+02	C16H32O3	Organic acids
380	1,3,4-Trihydroxy-5-oxocyclohexane-1-carboxylic acid	1.90E+02	C7H10O6	Organic acids

381	L-Aspartyl-L-Phenylalanine	2.80E+02	C13H16N2O5	Amino acids and derivatives
382	D-Threonic Acid	1.36E+02	C4H8O5	Others
383	Isoascorbic acid 2-O-glucoside	3.38E+02	C12H18O11	Others
384	Rutinose	3.26E+02	C12H22O10	Others
385	2,2-Dimethylsuccinic acid	1.46E+02	C6H10O4	Organic acids
386	Malonyltryptophan	2.90E+02	C14H14N2O5	Organic acids
387	N-(malonyl)phenylalanine	2.51E+02	C12H13NO5	Amino acids and derivatives
388	Glu-Ile-Glu	3.89E+02	C16H27N3O8	Amino acids and derivatives
389	2,3-Dihydroxypropanal	9.00E+01	C3H6O3	Others
390	L-Cycloserine	1.02E+02	C3H6N2O2	Amino acids and derivatives
391	Maltopentaose	8.28E+02	C30H52O26	Others
392	2-Hydroxy-4-methyl-3-tridecanoyloxypentanoic acid methyl ester	3.58E+02	C20H38O5	Lipids
393	DL-3-Phenyllactic acid	1.66E+02	C9H10O3	Organic acids
394	L-Homocitrulline	1.89E+02	C7H15N3O3	Amino acids and derivatives
395	1-O-Linoleoyl-(carbon eicosapentaenoic acid)	5.82E+02	C38H62O4	Lipids
396	δ-Guanidinovaleric acid	1.59E+02	C6H13N3O2	Organic acids
397	DMelezitose O-rhamnoside	6.50E+02	C24H42O20	Others
398	Glu-Phe-Ala	3.65E+02	C17H23N3O6	Amino acids and derivatives
399	D-Sedoheptuiose 7-phosphate	2.90E+02	C7H15O10P	Others
400	8-Azaguanine	1.52E+02	C4H4N6O	Nucleotides and derivatives
401	Erythorbic Acid; Isoascorbic Acid	1.76E+02	C6H8O6	Others
402	5-L-Glutamyl-L-amino acid	2.18E+02	C8H14N2O5	Amino acids and derivatives
403	D-Fucose	1.64E+02	C6H12O5	Others
404	3-Hydroxy-4H-pyran-4-one	1.12E+02	C5H4O3	Organic acids
405	2-Palmitoylglycerol	3.30E+02	C19H38O4	Lipids
406	Oxalacetic acid	1.32E+02	C4H4O5	Organic acids
407	Phosphoenolpyruvate	1.68E+02	C3H5O6P	Organic acids
408	D-Glucose	1.80E+02	C6H12O6	Others
409	3-Phospho-D-glyceric acid	1.86E+02	C3H7O7P	Others
410	Dihydroxyacetone phosphate	1.70E+02	C3H7O6P	Others
411	D-Glucose-1-phosphate*	2.60E+02	C6H13O9P	Others
412	α-Ketoglutaric acid	1.46E+02	C5H6O5	Organic acids
413	Fumaric acid	1.16E+02	C4H4O4	Organic acids
414	Succinic acid*	1.18E+02	C4H6O4	Organic acids
415	Citric Acid	1.92E+02	C6H8O7	Organic acids
416	L-Malic acid	1.34E+02	C4H6O5	Organic acids
417	Cis-Aconitic acid	1.74E+02	C6H6O6	Organic acids
418	Isocitric Acid	1.92E+02	C6H8O7	Organic acids
419	Ribulose-5-phosphate	2.30E+02	C5H11O8P	Others
420	2-Keto-3-deoxygluconate	1.78E+02	C6H10O6	Organic acids
421	2-Deoxyribose-1-phosphate	2.14E+02	C5H11O7P	Nucleotides and derivatives

422	DL-Glyceric Acid	1.06E+02	C3H6O4	Organic acids
423	D-Erythrose-4-phosphate	2.00E+02	C4H9O7P	Others
424	Gluconic acid	1.96E+02	C6H12O7	Others
425	D-Glucono-1,5-lactone*	1.78E+02	C6H10O6	Others
426	D-Ribose	1.50E+02	C5H10O5	Others
427	Uridine 5'-diphospho-D-glucose	5.66E+02	C15H24N2O17 P2	Nucleotides and derivatives
428	D-Glucoronic acid*	1.94E+02	C6H10O7	Others
429	D-Galacturonic acid*	1.94E+02	C6H10O7	Others
430	D-Arabinose	1.50E+02	C5H10O5	Others
431	D-Xylonic acid	1.66E+02	C5H10O6	Others
432	D-Arabitol	1.52E+02	C5H12O5	Others
433	Xylitol	1.52E+02	C5H12O5	Others
434	D-Mannose	1.80E+02	C6H12O6	Others
435	D-Sorbitol	1.82E+02	C6H14O6	Others
436	Sorbitol-6-phosphate	2.62E+02	C6H15O9P	Others
437	D-Mannitol	1.82E+02	C6H14O6	Others
438	Inositol	1.80E+02	C6H12O6	Others
439	D-Fructose 6-phosphate*	2.60E+02	C6H13O9P	Others
440	D-Sucrose	3.42E+02	C12H22O11	Others
441	N-Acetyl-D-galactosamine	2.21E+02	C8H15NO6	Others
442	D-Galactose	1.80E+02	C6H12O6	Others
443	Galactinol	3.42E+02	C12H22O11	Others
444	Dulcitol	1.82E+02	C6H14O6	Others
445	Stachyose	6.66E+02	C24H42O21	Others
446	4,5,6-Trihydroxy-2-oxohexanoic acid	1.78E+02	C6H10O6	Organic acids
447	Raffinose	5.04E+02	C18H32O16	Others
448	Melibiose	3.42E+02	C12H22O11	Others
449	Dehydroascorbic acid	1.74E+02	C6H6O6	Others
450	L-Ascorbic acid (Vitamin C)	1.76E+02	C6H8O6	Others
451	Tartronate semialdehyde	1.04E+02	C3H4O4	Organic acids
452	D-Saccharic acid	2.10E+02	C6H10O8	Others
453	3-Dehydro-L-Threonic Acid	1.34E+02	C4H6O5	Others
454	D-Glucurono-6,3-lactone	1.76E+02	C6H8O6	Others
455	D-Arabinono-1,4-lactone	1.48E+02	C5H8O5	Others
456	D-Galactaric acid	2.10E+02	C6H10O8	Others
457	2-Dehydro-3-deoxy-L-arabinonate	1.48E+02	C5H8O5	Others
458	L-Gulono-1,4-Lactone*	1.78E+02	C6H10O6	Others
459	Palmitic acid	2.56E+02	C16H32O2	Lipids
460	Decanoic acid	1.72E+02	C10H20O2	Organic acids
461	Dodecanoic acid (Lauric acid)	2.00E+02	C12H24O2	Lipids
462	Palmitaldehyde	2.40E+02	C16H32O	Lipids
463	10,16-Dihydroxypalmitic acid	2.88E+02	C16H32O4	Lipids
464	Hexadecanedioic acid	2.86E+02	C16H30O4	Lipids
465	L-Tyrosine	1.81E+02	C9H11NO3	Amino acids and derivatives
466	Delta-Tocopherol	4.02E+02	C27H46O2	Others
467	ATP; Adenosine 5'-Triphosphate	5.07E+02	C10H16N5O13 P3	Nucleotides and derivatives
468	Adenosine 5'-diphosphate	4.27E+02	C10H15N5O10 P2	Nucleotides and derivatives

469	Nicotinic acid adenine dinucleotide	6.63E+02	C21H27N7O14 P2	Nucleotides and derivatives
470	Flavin Single Nucleotide(FMN)	4.56E+02	C17H21N4O9P	Nucleotides and derivatives
471	NADP (Nicotinamide adenine dinucleotide phosphate)	7.43E+02	C21H28N7O17 P3	Nucleotides and derivatives
472	L-Glutamine	1.46E+02	C5H10N2O3	Amino acids and derivatives
473	L-Aspartic Acid	1.33E+02	C4H7NO4	Amino acids and derivatives
474	L-Arginine	1.74E+02	C6H14N4O2	Amino acids and derivatives
475	L-Ornithine	1.32E+02	C5H12N2O2	Amino acids and derivatives
476	N-α-Acetyl-L-ornithine	1.74E+02	C7H14N2O3	Amino acids and derivatives
477	N-Acetyl-L-glutamic acid	1.89E+02	C7H11NO5	Amino acids and derivatives
478	L-Citrulline	1.75E+02	C6H13N3O3	Amino acids and derivatives
479	Xanthine	1.52E+02	C5H4N4O2	Nucleotides and derivatives
480	Xanthosine	2.84E+02	C10H12N4O6	Nucleotides and derivatives
481	L-Glycine	7.50E+01	C2H5NO2	Amino acids and derivatives
482	Adenosine-5'-phosphosulfate	4.27E+02	C10H14N5O10 PS	Nucleotides and derivatives
483	Oxalic acid	9.00E+01	C2H2O4	Organic acids
484	Adenine	1.35E+02	C5H5N5	Nucleotides and derivatives
485	Adenosine 5'-monophosphate	3.47E+02	C10H14N5O7P	Nucleotides and derivatives
486	Oxamic acid	8.90E+01	C2H3NO3	Amino acids and derivatives
487	Guanosine 3',5'-cyclic monophosphate	3.45E+02	C10H12N5O7P	Nucleotides and derivatives
488	3'-Adenylic Acid	3.47E+02	C10H14N5O7P	Nucleotides and derivatives
489	Inosine 5'-monophosphate	3.48E+02	C10H13N4O8P	Nucleotides and derivatives
490	2'-Deoxyadenosine-5'-monophosphate	3.31E+02	C10H14N5O6P	Nucleotides and derivatives
491	Inosine diphosphate	4.28E+02	C10H14N4O11 P2	Nucleotides and derivatives
492	Hypoxanthine	1.36E+02	C5H4N4O	Nucleotides and derivatives
493	Guanine	1.51E+02	C5H5N5O	Nucleotides and derivatives
494	Guanosine 5'-monophosphate	3.63E+02	C10H14N5O8P	Nucleotides and derivatives

495	Adenosine	2.67E+02	C10H13N5O4	Nucleotides and derivatives
496	2'-Deoxyguanosine	2.67E+02	C10H13N5O4	Nucleotides and derivatives
497	Guanosine	2.83E+02	C10H13N5O5	Nucleotides and derivatives
498	2'-Deoxyadenosine*	2.51E+02	C10H13N5O3	Nucleotides and derivatives
499	2'-Deoxyinosine-5'-monophosphate	3.32E+02	C10H13N4O7P	Nucleotides and derivatives
500	Methylmalonic acid*	1.18E+02	C4H6O4	Organic acids
501	3-Hydroxypropanoic acid	9.00E+01	C3H6O3	Organic acids
502	3-Ureidopropionic Acid	1.32E+02	C4H8N2O3	Organic acids
503	Uracil	1.12E+02	C4H4N2O2	Nucleotides and derivatives
504	Uridine 5'-diphosphate	4.04E+02	C9H14N2O12P2	Nucleotides and derivatives
505	Barbituric acid;Malonylurea;2,4,6-Pyrimidinetrione	1.28E+02	C4H4N2O3	Nucleotides and derivatives
506	5-Methylcytosine	1.25E+02	C5H7N3O	Nucleotides and derivatives
507	β-Pseudouridine	2.44E+02	C9H12N2O6	Nucleotides and derivatives
508	Cytosine	1.11E+02	C4H5N3O	Nucleotides and derivatives
509	2'-Deoxycytidine-5'-monophosphate	3.07E+02	C9H14N3O7P	Nucleotides and derivatives
510	Cytidine 5'-monophosphate(Cytidylic acid)	3.23E+02	C9H14N3O8P	Nucleotides and derivatives
511	Uridine 5'-monophosphate	3.24E+02	C9H13N2O9P	Nucleotides and derivatives
512	Uridine	2.44E+02	C9H12N2O6	Nucleotides and derivatives
513	Cytidine	2.43E+02	C9H13N3O5	Nucleotides and derivatives
514	2'-Deoxycytidine	2.27E+02	C9H13N3O4	Nucleotides and derivatives
515	Orotic acid (Vitamin B13)	1.56E+02	C5H4N2O4	Others
516	γ-Aminobutyric acid	1.03E+02	C4H9NO2	Organic acids
517	Succinic semialdehyde	1.02E+02	C4H6O3	Organic acids
518	N-Acetyl-L-Aspartic Acid	1.75E+02	C6H9NO5	Amino acids and derivatives
519	L-Threonine	1.19E+02	C4H9NO3	Amino acids and derivatives
520	L-Homoserine	1.19E+02	C4H9NO3	Amino acids and derivatives
521	Creatine	1.31E+02	C4H9N3O2	Organic acids
522	L-Tryptophan	2.04E+02	C11H12N2O2	Amino acids and derivatives
523	Hydroxypyruvic acid	1.04E+02	C3H4O4	Organic acids

524	N,N-Dimethylglycine	1.03E+02	C4H9NO2	Amino acids and derivatives
525	S-(5'-Adenosyl)-L-methionine	3.98E+02	C15H22N6O5S	Amino acids and derivatives
526	N-Formyl-L-Methionine	1.77E+02	C6H11NO3S	Amino acids and derivatives
527	L-Homocystine	2.68E+02	C8H16N2O4S2	Amino acids and derivatives
528	L-Methionine	1.49E+02	C5H11NO2S	Amino acids and derivatives
529	Glutathione reduced form	3.07E+02	C10H17N3O6S	Amino acids and derivatives
530	O-Acetylserine	1.47E+02	C5H9NO4	Amino acids and derivatives
531	5'-Deoxy-5'-(methylthio)adenosine	2.97E+02	C11H15N5O3S	Nucleotides and derivatives
532	L-Homocysteine	1.35E+02	C4H9NO2S	Amino acids and derivatives
533	L-Methionine Sulfoxide	1.65E+02	C5H11NO3S	Amino acids and derivatives
534	S-Ribosyl-L-homocysteine	2.67E+02	C9H17NO6S	Amino acids and derivatives
535	S-(5'-Adenosy)-L-homocysteine	3.84E+02	C14H20N6O5S	Amino acids and derivatives
536	L-Valine	1.17E+02	C5H11NO2	Amino acids and derivatives
537	L-Isoleucine*	1.31E+02	C6H13NO2	Amino acids and derivatives
538	L-Leucine*	1.31E+02	C6H13NO2	Amino acids and derivatives
539	β-Hydroxyisovaleric acid	1.18E+02	C5H10O3	Organic acids
540	2-Isopropylmalic Acid	1.76E+02	C7H12O5	Organic acids
541	2-Hydroxy-2-methyl-3-oxobutanoic acid*	1.32E+02	C5H8O4	Organic acids
542	3-Methylmalic acid	1.48E+02	C5H8O5	Organic acids
543	3-Isopropylmalic Acid*	1.76E+02	C7H12O5	Organic acids
544	L-Lysine	1.46E+02	C6H14N2O2	Amino acids and derivatives
545	Allysine(6-Oxo DL-Norleucine)	1.45E+02	C6H11NO3	Amino acids and derivatives
546	L-Saccharopine	2.76E+02	C11H20N2O6	Amino acids and derivatives
547	2,6-Diaminooimelic acid	1.90E+02	C7H14N2O4	Organic acids
548	Homocitrate	2.06E+02	C7H10O7	Organic acids
549	L-Pipecolic Acid	1.29E+02	C6H11NO2	Organic acids
550	Trimethyllysine	1.88E+02	C9H20N2O2	Amino acids and derivatives
551	5-Acetamidopentanoic Acid	1.59E+02	C7H13NO3	Organic acids
552	N6-Acetyl-L-lysine	1.88E+02	C8H16N2O3	Amino acids and derivatives
553	L-Proline	1.15E+02	C5H9NO2	Amino acids and derivatives

554	Trans-4-Hydroxy-L-proline*	1.31E+02	C5H9NO3	Amino acids and derivatives
555	4-Guanidinobutyric acid	1.45E+02	C5H11N3O2	Organic acids
556	N-carbamoylputrescine	1.31E+02	C5H13N3O	Amino acids and derivatives
557	Creatinine	1.13E+02	C4H7N3O	Organic acids
558	L-Histidine	1.55E+02	C6H9N3O2	Amino acids and derivatives
559	Urocanic acid	1.38E+02	C6H6N2O2	Organic acids
560	3,4-Dihydroxy-L-phenylalanine (L-Dopa)	1.97E+02	C9H11NO4	Amino acids and derivatives
561	Phenylacetyl-L-glutamine	2.64E+02	C13H16N2O4	Amino acids and derivatives
562	L-Phenylalanine	1.65E+02	C9H11NO2	Amino acids and derivatives
563	Phenylpyruvic acid	1.64E+02	C9H8O3	Organic acids
564	N-Acetyl-L-phenylalanine	2.07E+02	C11H13NO3	Amino acids and derivatives
565	4-Hydroxy-2-Oxopentanoic Acid*	1.32E+02	C5H8O4	Organic acids
566	5-Hydroxy-L-tryptophan	2.20E+02	C11H12N2O3	Amino acids and derivatives
567	2-Picolinic acid	1.23E+02	C6H5NO2	Organic acids
568	D-Fructose-1,6-biphosphate	3.40E+02	C6H14O12P2	Others
569	Shikimic acid	1.74E+02	C7H10O5	Organic acids
570	3-Dehydroshikimic acid	1.72E+02	C7H8O5	Organic acids
571	Quinic Acid	1.92E+02	C7H12O6	Organic acids
572	N-acetyl-beta-alanine	1.31E+02	C5H9NO3	Amino acids and derivatives
573	D-Pantothenic Acid	2.19E+02	C9H17NO5	Others
574	2-Aminoethanesulfonic acid	1.25E+02	C2H7NO3S	Organic acids
575	2-Aminoethanesulfinic acid	1.09E+02	C2H7NO2S	Organic acids
576	2-Hydroxyethylphosphonic acid	1.26E+02	C2H7O4P	Organic acids
577	Pyrrole-2-carboxylic acid	1.11E+02	C5H5NO2	Organic acids
578	cis-4-Hydroxy-D-proline*	1.31E+02	C5H9NO3	Amino acids and derivatives
579	5-Oxoproline*	1.29E+02	C5H7NO3	Amino acids and derivatives
580	L-Cysteinyl-L-glycine	1.78E+02	C5H10N2O3S	Amino acids and derivatives
581	5-Oxo-L-Proline*	1.29E+02	C5H7NO3	Amino acids and derivatives
582	Oxiglutatione	6.12E+02	C20H32N6O12 S2	Amino acids and derivatives
583	D-Glucose 6-phosphate*	2.60E+02	C6H13O9P	Others
584	D-Glucose 1,6-bisphosphate	3.40E+02	C6H14O12P2	Others
585	Trehalose 6-phosphate	4.22E+02	C12H23O14P	Others
586	D-Trehalose	3.42E+02	C12H22O11	Others
587	Keto-Deoxy-Nonulonic acid	2.68E+02	C9H16O9	Organic acids
588	N-Acetyl-D-mannosamine	2.21E+02	C8H15NO6	Others
589	D-Glucosamine 1-phosphate	2.59E+02	C6H14NO8P	Others
590	Phytic acid	6.60E+02	C6H18O24P6	Organic acids

591	Choline Alfoscerate	2.57E+02	C8H20NO6P	Lipids
592	5,6-DiHETrE[(±)5,6-dihydroxy-8Z,11Z,14Z-eicosatrienoic acid]	3.38E+02	C20H34O4	Lipids
593	5S,8R-DiHODE; (5S,8R,9Z,12Z)-5,8-Dihydroxyoctadeca-9,12-dienoate	3.12E+02	C18H32O4	Lipids
594	13(S)-HODE;13(S)-Hydroxyoctadeca-9Z,11E-dienoic acid*	2.96E+02	C18H32O3	Lipids
595	9,10,13-Trihydroxy-11-Octadecenoic Acid	3.30E+02	C18H34O5	Lipids
596	9S-Hydroxy-10E,12Z-octadecadienoic acid*	2.96E+02	C18H32O3	Lipids
597	7S,8S-DiHODE; (9Z,12Z)-(7S,8S)-Dihydroxyoctadeca-9,12-dienoic acid	3.12E+02	C18H32O4	Lipids
598	9,10-Dihydroxy-12,13-epoxyoctadecanoic acid	3.30E+02	C18H34O5	Lipids
599	(9Z,11E)-Octadecadienoic acid*	2.80E+02	C18H32O2	Lipids
600	Crepenynic acid	2.78E+02	C18H30O2	Lipids
601	9,12,13-TriHOME; 9(S),12(S),13(S)-Trihydroxy-10(E)-octadecenoic acid	3.30E+02	C18H34O5	Lipids
602	12,13-DHOME; (9Z)-12,13-Dihydroxyoctadec-9-enoic acid	3.14E+02	C18H34O4	Lipids
603	9-Oxo-10E,12Z-octadecadienoic acid	2.94E+02	C18H30O3	Lipids
604	Linoleic acid*	2.80E+02	C18H32O2	Lipids
605	γ-Linolenic Acid	2.78E+02	C18H30O2	Lipids
606	9S-Hydroperoxy-10E,12Z-octadecadienoic acid	3.12E+02	C18H32O4	Lipids
607	13S-Hydroperoxy-9Z,11E-octadecadienoic acid	3.12E+02	C18H32O4	Lipids
608	9(10)-EpOME;(9R,10S)-(12Z)-9,10-Epoxyoctadecenoic acid	2.96E+02	C18H32O3	Lipids
609	12,13-Epoxy-9-Octadecenoic Acid	2.96E+02	C18H32O3	Lipids
610	2R-hydroxy-9Z,12Z,15Z-octadecatrienoic acid	2.94E+02	C18H30O3	Lipids
611	17-Hydroxylinolenic acid	2.94E+02	C18H30O3	Lipids
612	13S-Hydroxy-9Z,11E,15Z-octadecatrienoic acid	2.94E+02	C18H30O3	Lipids
613	α-Linolenic Acid	2.78E+02	C18H30O2	Lipids
614	13(s)-hydroperoxy-(9z,11e,15z)-octadecatrienoic acid	3.10E+02	C18H30O4	Lipids
615	Jasmonic acid	2.10E+02	C12H18O3	Organic acids
616	9-Hydroperoxy-10E,12,15Z-octadecatrienoic acid	3.10E+02	C18H30O4	Lipids
617	9-Hydroxy-12-oxo-15(Z)-octadecenoic acid	3.12E+02	C18H32O4	Lipids
618	9-Hydroxy-12-oxo-10(E),15(Z)-octadecadienoic acid	3.10E+02	C18H30O4	Lipids
619	Methyl jasmonate	2.24E+02	C13H20O3	Organic acids
620	2-Dodecenedioic acid	2.28E+02	C12H20O4	Lipids
621	D-Sphingosine	2.99E+02	C18H37NO2	Lipids
622	Dihydrosphingosine	3.01E+02	C18H39NO2	Lipids
623	Dihydrosphingosine-1-Phosphate	3.81E+02	C18H40NO5P	Lipids
624	2-Propylmalic Acid*	1.76E+02	C7H12O5	Organic acids
625	D-Lactic Acid	9.00E+01	C3H6O3	Organic acids
626	L-threo-3-Methylaspartate	1.47E+02	C5H9NO4	Amino acids and derivatives
627	L-Tartaric acid	1.50E+02	C4H6O6	Organic acids
628	3-Hydroxybutyric acid	1.04E+02	C4H8O3	Organic acids
629	2-Methyl-3-oxosuccinic acid	1.46E+02	C5H6O5	Organic acids
630	L-Citramalic acid	1.48E+02	C5H8O5	Organic acids
631	Itaconic acid	1.30E+02	C5H6O4	Organic acids
632	Sedoheptulose	2.10E+02	C7H14O7	Others
633	Riboflavin 5'-Adenosine Diphosphate	7.85E+02	C27H33N9O15 P2	Nucleotides and derivatives
634	Riboflavin (Vitamin B2)	3.76E+02	C17H20N4O6	Others
635	4-Pyridoxic acid	1.83E+02	C8H9NO4	Others

636	Pyridoxine	1.69E+02	C8H11NO3	Others
637	Pyridoxal	1.67E+02	C8H9NO3	Others
638	Pyridoxine-5'-phosphate	2.49E+02	C8H12NO6P	Others
639	Nicotinic acid (Vitamin B3)	1.23E+02	C6H5NO2	Others
640	β-Nicotinamide mononucleotide	3.34E+02	C11H15N2O8P	Nucleotides and derivatives
641	Nicotinate D-ribonucleoside	2.56E+02	C11H14NO6+	Others
642	Nicotinamide	1.22E+02	C6H6N2O	Others
643	Biotin	2.44E+02	C10H16N2O3S	Others
644	Mevalonic acid	1.48E+02	C6H12O4	Organic acids
645	Abscisic acid	2.64E+02	C15H20O4	Organic acids
646	Riboprine	3.35E+02	C15H21N5O4	Nucleotides and derivatives
647	Methanesulfonic acid	9.60E+01	CH4O3S	Organic acids
648	Cis-4,7,10,13,16,19-Docosahexaenoic Acid	3.28E+02	C22H32O2	Lipids
649	Eicosadienoic acid	3.08E+02	C20H36O2	Lipids
650	Erucic acid	3.38E+02	C22H42O2	Lipids
651	6-Aminocaproic acid	1.31E+02	C6H13NO2	Organic acids
652	Adipic Acid*	1.46E+02	C6H10O4	Organic acids
653	4-Hydroxyretinoic acid	3.16E+02	C20H28O3	Organic acids
654	Muconic acid	1.42E+02	C6H6O4	Organic acids
655	N-Methyl-L-Glutamate	1.61E+02	C6H11NO4	Amino acids and derivatives
656	2-Furoic acid	1.12E+02	C5H4O3	Organic acids
657	S-Methyl-L-cysteine	1.35E+02	C4H9NO2S	Amino acids and derivatives
658	Maltotriose	5.04E+02	C18H32O16	Others
659	Jasmonoyl-L-Isoleucine	3.23E+02	C18H29NO4	Amino acids and derivatives
